# circ_0000045 promotes proliferation, migration, and invasion of head and neck squamous cell carcinomas via regulating HSP70 and MAPK pathway

**DOI:** 10.1186/s12885-022-09880-y

**Published:** 2022-07-20

**Authors:** Ronghao Sun, Yuqiu Zhou, Yongcong Cai, Chunyan Shui, Xu Wang, Jingqiang Zhu

**Affiliations:** 1 Department of Head and Neck Surgery, Sichuan Cancer Hospital and Institute,University of Electronic Science and Technology of China, No.55, 4th section of Southern Renmin Road, Chengdu, Sichuan 610041 China; 2 Department of Thyroid and Parathyroid Surgery, West China Hospital, No. 37, Guoxue Alley, Chengdu, Sichuan 610041 China

**Keywords:** circ_0000045, circRNA, HNSCC, HSP70, MAPK, JNK2, P38

## Abstract

**Objective:**

Head and neck squamous cell carcinoma (HNSCC) is one severe malignancy driven by complex cellular and signaling mechanisms. However, the roles of circular RNAs (circRNAs) in HNSCC’s development remains poorly understood. Therefore, this study investigated the functions of differentially expressed circRNAs in regulating HNSCC cell functions.

**Methods:**

Differentially expressed circRNAs were characterized through RNA sequencing in HNSCC tissues. CircRNA’s identity was then confirmed using RT-PCR and Sanger’s sequencing. Next, expression levels of circRNA and mRNA were detected by qRT-PCR, after which protein abundances were measured by Western blotting. Subsequently, the proliferation, migration, and invasion of HNSCC cells was assessed by MTS, wound healing, and Transwell system, respectively, followed by identification of circRNA-binding proteins in HNSCC cells by circRNA pull-down, coupled with mass spectrometry.

**Results:**

Great alterations in circRNA profiles were detected in HNSCC tissues, including the elevated expression of circ_0000045. As observed, silencing of circ_0000045 effectively repressed the proliferation, migration, and invasion of HNSCC cell lines (FaDu and SCC-9). Contrarily, circ_0000045’s overexpression promoted the proliferation, migration, and invasion in FaDu and SCC-9 cells. Results also showed that circ_0000045 was associated with multiple RNA-binding proteins in HNSCC cells, such as HSP70. Moreover, circ_0000045 knockdown enhanced HSP70 expression and inhibited JNK2 and P38’s expression in HNSCC cells, which were oppositely regulated by circ_0000045’s overexpression.

**Conclusion:**

The high expression of circ_0000045; therefore, promoted cell proliferation, migration, and invasion during HNSCC’s development through regulating HSP70 protein and mitogen-activated protein kinase signaling.

**Supplementary Information:**

The online version contains supplementary material available at 10.1186/s12885-022-09880-y.

## Introduction

Head and neck squamous cell carcinoma (HNSCC) is one common and severe malignant disease whose incidence continues to rise. It has been anticipated that an average of 1.08 million new cases will occur annually by 2030 [1]. Poor prognosis, high lymph node metastasis, and increased mortality rates are among the characteristics of HNSCC [2, 3]. Moreover, its initiation has been associated with multiple etiological factors, such as cigarette smoking, alcohol consumption, and human papillomavirus infection [1, 4]. Furthermore, the pathogenesis of HNSCC has been proposed to be mediated by serial molecular events like CD44, CD133, and ALDH1 [5–7]. Although the recent clinical management of patients with HNSCC mainly depends on surgical procedures, chemo- and radiotherapy, and immunotherapy, such as PD1, PDL1, and epidermal growth factor receptor (EGFR) inhibitors [1, 8], in addition to the significant progress recorded in diagnosis and treatment during the past decades, the survival rates for HNSCC patients remains low. This low survival rate is mainly due to late diagnosis, high recurrence rate, and poor prognosis [1]. Therefore, the development of new preventive and therapeutic strategies for HNSCC patients should be based on the further elucidation of pathogenic molecular mechanisms.

Non-coding RNAs (ncRNAs) have been characterized as essential players in HNSCC’s development in the past decades because of their prevalent involvements in regulating HNSCC cells’ survival, proliferation, metabolism, invasion, and tumor microenvironment remodeling [9]. For instance, the microRNA miR-31 and its suppression of SIRT3 (Silent information regulator 3) gene expression mediated the disruption of mitochondrial activity and membrane integrity, including the burst in oxidative stress and metabolic aberrances during HNSCC’s progression [10]. As another significant kind of bioactive ncRNAs, circular RNAs (circRNAs) refer to a large group of highly conserved ncRNAs, having a closed and single-stranded loop structure, which are formed via reverse splicing and are tissue-specifically expressed, Some of their information can be found in circBase (http://circbase.org/), CIRCpedia (https://www.picb.ac.cn/rnomics/circpedia/), CircInteractome (https://circinteractome.irp.nia.nih.gov/), and deepbase (http://deepbase.sysu.edu.cn/) [9, 11]. Recent extensive research has also shown that circRNAs perform their biological and pathogenic roles mainly through interfering genes’ expression, sponging microRNAs or proteins, and producing small functional peptides [9]. Moreover, several circRNAs were identified as novel biomarkers for HNSCC’s early diagnosis and pathogenic staging [11]. Furthermore, circRNAs can regulate multiple cancer-related signaling pathways, such as the microRNA sponges during HNSCC’s pathogenesis, involving EGFR, Notch, mTOR (mammalian target of Rapamycin), and MAPK (mitogen-activated protein kinase) signaling pathways [11–14]. Among them, the circRNA CiRS-7 was recently reported to enhance the metastasis of HNSCC by modulating the MAPK and AKT (protein kinase B) signaling cascades via sponging RAF-1 mRNAs [12]. Therefore, even though specific roles and underlying mechanisms of circRNAs are involved in HNSCC’s pathogenesis, further explorations are required.

RNA-binding proteins (RBPs) typically refer to proteins capable of binding RNA molecules via their RBDs (RNA-binding domains), resulting in functional alterations or stability of bound RNA molecules [15, 16]. Recent research revealed that some RNAs bound with target proteins to regulate biological functions, stabilities, subcellular localization, or interactions with other partners [16]. For instance, the circRNA; circFOXK2, which was highly expressed in PDAC (pancreatic ductal adenocarcinoma) tissue samples, promoted the proliferation and invasion of PDAC cells by interacting with RBPs, YBX1 (Y-box binding protein 1), and hnRNPK (heterogeneous nuclear ribonucleoprotein K), to enhance the expression of oncogenes [17]. Furthermore, the proto-oncogenic or tumor-suppressing roles of RBPs were widely observed in various human cancers, showing the prevalent involvements of RBPs in cancer development mediated by the sustenance of cell proliferation, evasion of cell death, and regulation of migration, invasion, and angiogenesis [18]. Moreover, multiple RBPs, such as ADAR1 (adenosine deaminase acting on RNA 1) and DDX3 (DEAD-box RNA helicase 3) [19], were reported to be differentially expressed during HNSCC’s development and metastasis. Specifically, another RNA-binding protein, FXR1 (fragile X-related protein 1), bypassed the P53-induced HNSCC cell’s senescence via destabilizing p21 mRNA. However, stabilizing of the non-coding RNA, TERC (Telomerase RNA Component) was observed [20]. Yet, little is known about the interaction of RBPs with circRNAs associated with HNSCC’s development and progression.

Therefore we conducted a large-scale screening of circRNAs differentially expressed in HNSCC tissue samples in this study to investigate the potential association of circRNA with HNSCC’s pathogenesis. Furthermore, the roles of the identified circRNA molecules in regulating HNSCC’s cell proliferation, migration, and invasion were explored further, focusing on its interaction with RBPs. Results are proposed to provide novel insights into the molecular mechanisms underlying HNSCC’s development based on circRNA-RBP’s interaction, which can also serve as a basis for early cancer diagnosis and new therapeutic drug development.

## Material and methods

### Patients and tissue collection

Total 14 patients diagnosed with oral tongue squamous cell carcinoma that had been treated using surgical section at the Department of Head and Neck Surgery, Sichuan Cancer Hospital, Electronic Science and Technology University (Sichuan, China) between January 2019 and December 2021, were enrolled in this study. Detailed clinicopathological characteristics of these patients are shown in supplemental Table 1. The Ethics Committee of the Sichuan Cancer University approved all experiments involving clinical samples in advance (approval number: 20201120). Furthermore, before surgical operations, each patient signed the written informed consent. Then, HNSCC tissue samples and corresponding adjacent non-cancerous tongue tissue samples were collected from the patients mentioned above by surgery and immediately stored in liquid nitrogen for subsequent assays.

### RNA sequencing

Analysis of circular RNA profiles in HNSCC and adjacent normal tongue tissue samples were conducted by deep RNA sequencing as previously described with minor modifications [21]. Briefly, total RNA samples were isolated from obtained tongue tissues using the TRIzol^TM^ reagent (#15596026; Thermo Fisher Scientific), following the manufacturer’s instructions. Then, size distribution and integrity of RNA samples from tongue tissues were assessed using Agilent 2100 Bioanalyzer and agarose gel electrophoresis, followed with the removal of rRNA constitutes using the TruSeq RNA Sample Preparation Kit (Illumina, USA). Afterward, an RNA library was established from similar volumes of RNA samples in each group using the NEBNext® RNA Library Prep Kit for Illumina (##E7775; NEB) according to the manufacturer’s instructions, after which they were subjected to deep sequencing on a HiseqTM 2000 system (Illumina, USA).

The alignment of clean reads was conducted using the Bowtie2 method (bowtie-bio.sourceforge.net/bowtie2). First, we selected read junctions and finished the prediction and annotation of circular RNA using the CIRI software [22]. Then, relative expressional levels of circRNAs were measured by calculating RPM (Mapped back splicing junction reads per million mapped reads). Significantly differentially expressed circRNAs between the normal and HNSCC tongue tissues were defined by a fold change of >= 1.5 or <= 0.67 and a *P*-value of < 0.05. Finally, hierarchical clustering of differentially expressed circular RNAs was constructed with a heatmap and volcano plot using RStudio software (version 3.5.1). For the volcano plot, when the circRNA’s expression level was 0, we assumed 0 as 0.000000001 to calculate the value of Log10TPM. However, the identity of target circular RNAs was validated by RT-PCR and Sanger’s DNA sequencing.

### RT-PCR and qRT-PCR

Total RNA samples from tissues and cell lines were prepared and quantified as described above. From the obtained RNA samples, 1.8 μg RNA sample was used for cDNA synthesis using the Bestar RT kit (#2220; DBI Bioscience) following the manufacturer’s instructions. Subsequently, while PCR analysis using divergent primers was conducted to verify the identity of characterized circRNAs, corresponding convergent primers targeting linear RNA molecules were used as negative controls. GAPDH was also detected during RT-PCR as the control group. Finally, relative expressional levels of circRNAs and mRNAs in tissues or cell lines were measured via real-time quantitative PCR, using the SYBR Green PCR Kit (#204057; Qiagen) according to the manufacturer’s instructions. Three biological replicates were conducted for circRNA and mRNA quantitation via the standard 2^-△△Ct^ method, using the GAPDH as an internal standard. Additionally, primer sequences used for RT-PCR and qRT-PCR are listed in Table 1.

**Table 1:** Information of primers used for PCR assay

Primer name	Primer sequences (5'-3')	Amplified sequence (5'-3')	Product size (bp)
Circ-0000045_↔-F	CGATGGCCTACTGGGGA	CGATGGCCTACTGGGGATAACATTCATGCAGAACATCAGGTGTTGGTGGAATGAGCGTTGCATGTGTCTTGAAGAGAAAAGCAGTGCTTTGGCAGGACTCTTTCAGCCCCCACCT	115
Circ-0000045_↔-R	AGGTGGGGGCTGAAAGAG		
Circ-0000045_→←-F	TGAAACATCACCCTCAAGAA	TGAAACATCACCCTCAAGAACCAGCTAATCCCAACATGCCTGTTGTTTTGACATCTGGAACAGGGTCGCAAGCGCAGCCACAACCAGCTGCAAATCAGGCTCTTGCAGCTGGGACTCACTCCAGCCCTGTCCCAGGATCTATAGGAGTTGCAGGCCGTTCCCAGGACGACGCTATGGTGGACTACTTCTTTCAGAGGCAGCATGGTGAGCAGCTTGGGGGAGGAGGAAGTGGAGGAGGCGGCTATAATAATAGCAAACATCGATGGCCTACTGGGGATAACATTC	285
Circ-0000045_→←-R	GAATGTTATCCCCAGTAGGC		
Circ-0008386_↔-F	TCGTCCTTATCTGGGAGTTGAG	TCGTCCTTATCTGGGAGTTGAGGATGCATTGTCCATTCGAAGTGTTGGCAGTCACAGGGGCTGAAAACACAGTTAAATCACTTTCAAAGTTAAAAGACTATTAAGAACACAAGATGGGGACTCCTGCTTCTGGAAGGAAAAGAACACCTGTGAAAGACCGATTTT	165
Circ-0008386_↔-R	AAAATCGGTCTTTCACAGGTGT		
Circ-0008386_→←-F	AGAGGCAAGGCTGGCAG	AGAGGCAAGGCTGGCAGCAAAACGGGCTGCCCGGGCAGAAGCAAGAGATATACGCATGAGAGAACTGGAACGACAACAAAAAGAGTACTCTCTTCATTCCTTTGATCGGAAGTGGGGACAGATTCAGAAGTGGCTGGAAGATTCGGAAAGGGCCAGGTATTCCCACCGGTCCAGTCACCATCGTCCTTA	189
Circ-0008386_→←-R	TAAGGACGATGGTGACTGGAC		
Circ-0004949_↔-F	CATGGTGGAGTATGTGTTGAG	CATGGTGGAGTATGTGTTGAGCTCATCCCCGGGCGATTCCTGTCTAAGAAAAGGAGGATTTCAGCTCTTGCCAGGTAAAAAGTTTTGGGAAACAGATGAATCCAGCAAAGATGGACCAA	119
Circ-0004949_↔-R	TTGGTCCATCTTTGCTGG		
Circ-0004949_→←-F	ACATCAGATCATTCAGTTTCCC	ACATCAGATCATTCAGTTTCCCAGCCAATCATGGTGCAGAGAAGACCTGGTCAGAGTTTCCATGTGAACAGTGAGGTCAATTCTGTACTGTCCCCACGATCGGAGAGTGGGGGACTAGGCGTTAGCATGGTGGAGTATGTGTTGAGCTCATCCCCGGGCGATTCCTGTCTAAGAAAAGGAGGATTT	186
Circ-0004949_→←-R	AAATCCTCCTTTTCTTAGACAGG		
Circ-0000043_↔-F	GGTACCTGTGGACTCAGCA	GGTACCTGTGGACTCAGCAGCAGCAACTGTGGGACTTTTTGACTACAATTCTCAACAACAGGGCCCAAGGGATGCAGACAGTGATGAAAACGACAAAGGTGAAAAGAAGAACAAGGGTACG	121
Circ-0000043_↔-R	CGTACCCTTGTTCTTCTTTTC		
Circ-0000043_→←-F	ATGGTTCTGAGGGCTTAGC	ATGGTTCTGAGGGCTTAGCCCAGCTGACCAGCACCAATGGTGCCAAGCCTGTGGAGGATTTCTCCAACATGGAGTCCCAGAGTGTCCCCTTGGACCCCATGGAACATGTGGGCATGGAGCCTCTTCAGTTTGATTATTCAGGCACGCAGGTACCTGTGGACTCAGCAGCAGCAACTGTGGGACTTTTTG	189
Circ-0000043_→←-R	CAAAAAGTCCCACAGTTGC		
HSP70-F	GAGCCAATGGAAACAGATCAGAAT	GAGCCAATGGAAACAGATCAGAATGCAAAGGAGGAAGAGAAGATGCAAGTGGACCAGGAGGAACCACATGTTGAAGAGCAACAGCAGCAGACACCAGCAGAAAATAAGGCAGAGTCTGAAGAAATGGAGACCTCTCAAGCTGGATCCAAGGATAAAAAGATGGACCAACCACCC	174
HSP70-R	GGGTGGTTGGTCCATCTTTTTAT		
HSPA8-F	GTCGCCTTTACGGACACTGAAC	GTCGCCTTTACGGACACTGAACGGTTGATCGGTGATGCCGCAAAGAATCAAGTTGCAATGAACCCCACCAACACAGTTTTTGATGCCAAACGTCTGATTGGACGCAGATTTGATGATGCTGTTGTCCAGTCTGATATGAAACATTGGCCCTTTATGGTGGTGAATGATGCTGGCAGGCCCAAGGTCCAAGTAGAATACAAGGGAGAG	207
HSPA8-R	GTCTCCCTTGTATTCTACTTGGACCT		
GAPDH(→←)-F	GAGTCAACGGATTTGGTCGT	GAGTCAACGGATTTGGTCGTATTGGGCGCCTGGTCACCAGGGCTGCTTTTAACTCTGGTAAAGTGGATATTGTTGCCATCAATGACCCCTTCATTGACCTCAACTACATGGTTTACATGTTCCAATATGATTCCACCCATGGCAAATTCCATGGCACCGTCAAGGCTGAGAACGGGAAGCTTGTC	185
GAPDH(→←)-R	GACAAGCTTCCCGTTCTCAG		
GAPDH(↔)-F	GTTCCAATATGATTCCACCCA	GTTCCAATATGATTCCACCCATGGCAAATTCCATGGCACCGTCAAGGCTGAGAACGGGAAGCTTGTCGAGTCAACGGATTTGGTCGTATTGGGCGCCTGGTCACCAGGGCTGCTTTTAACTCTGGT (Hypothetical)	
GAPDH(↔)-R	ACCAGAGTTAAAAGCAGCCCT		126

↔: divergent primers; →←: convergent primers

### Cell lines and transfection

HNSCC cell lines, Cal-27 (isolated from a tissue taken before treatment from a 56-year-old, white male with a lesion at the middle of the tongue in 1982), FaDu (separated from the pharynx of a 56-year-old male), SCC-4 (isolated from the tongue of a 55-year-old male), SCC-9 (isolated from the tongue of a 25-year-old male), and Detroit 562 (separated from the pharynx of a female pharyngeal cancer Caucasian patient) were purchased from the Cell Bank of the Chinese Academy of Sciences (Shanghai, China). First, cellular identities were confirmed through the Short Tandem Repeat profiling assay and cultured in DMEM (Dulbecco's modified eagle medium; Gibco) containing penicillin, streptomycin, and 10% FBS (Gibco) at 37°C in a standard cell culture chamber with 5% supply of CO_2_. Then, siRNA sequences of si-circ_0000045, si-circ_0008386, and the negative control (NC) were synthesized by the Genepharma company (Shanghai, China) and introduced into HNSCC cells using the Lipofectamine 3000 system (Thermo Fisher Scientific), following the manufacturer’s instructions to silencing circRNA expression. The siRNA sequences are showed in Table 2. Subsequently, the circ_0000045 sequence synthesized by a Genepharma company was ligated with the pcDNA3.1(+) vector-with flank sequence (General Bio, Anhui, China) to construct circ_0000045 overexpression plasmids, which were then transfected into HNSCC cells as introduced above to build the HNSCC OE-circ_0000045.

**Table 2:** siRNA sequences used in this study

Name of siRNA	Sequence (5'-3')
si-circ_0000045-1	AACAUCAGGUGUUGGUGGAAU
si-circ_0000045-2	GAACAUCAGGUGUUGGUGGAA
si-circ_0000045-3	UGCAGAACAUCAGGUGUUGGU
si-circ_0008386-1	GUCACAGGGGCUGAAAACACA
si-circ_0008386-2	GGCAGUCACAGGGGCUGAAAA
si-circ_0008386-3	ACAGGGGCUGAAAACACAGUU
negative control	UUCUCCGAACGUGUCACGUdTdT
si-HSP70	CCACUCUGCUUAUCAAGUUUCUGdTdT

### Cell viability

Viabilities of HNSCC cell lines were evaluated by the MTS method using a commercially available kit (#ab197010; Abcam) following the manufacturer’s instructions. Briefly, HNSCC cells cultured in 96-well plates (1 × 10^4^ cells per well) were incubated with MTS solutions (20 μl per well) at 37°C for 1.5 h-2 h under standard culture conditions with gentle shaking, which was then subjected to absorbance measurements at 490 nm (OD490) in a microplate reader. Finally, HNSCC proliferation rates were assessed with OD490 cells based on at least three biological replicates.

### Cell migration and invasion

Migration rates of HNSCC cells were assessed through the wound-healing method. Briefly, a monolayer containing HNSCC cells was produced using cultured 24-well plates for 24 h-48 h at 37°C under standard culture conditions. Before scratching, mitomycin C (10 μg/ml) was used to treat cells for three hours to inhibit proliferation. At the middle of 24-well plates, a straight scratching line was gently made using a 200 μl sterile pipette tip. Then, the normal culture medium was removed, washing was done thrice using phosphate-buffered saline, followed by the final addition of serum-free culture medium. Subsequently, scratching widths were measured after culturing at 37°C for 24 h and 48 h. Next, the migration rate was calculated as follows:

$$\mathrm{Migration}\ \mathrm{Rate}=\mathrm{migration}\ \mathrm{distance}\ \mathrm{at}\ 24\mathrm{h}\ \left(\mathrm{or}\ 48\ \mathrm{h}\right)/\mathrm{scratch}\ \mathrm{distance}\ \left(0\ \mathrm{h}\right)$$

Additionally, HNSCC cell invasion capabilities were evaluated using the Transwell system. Briefly, the inner walls of Transwell plates were pre-layered with Matrigel Matrix (BD Biocoat). Then, while HNSCC cells cultured in serum-free DMEM medium (1 × 10^6^ cells/ml) were added to the upper chambers of Transwell plates, the lower chambers were full of DMEM medium containing 10% FBS. After culturing at 37°C under standard culture conditions for 24 h–48 h, the HNSCC cells migrated to the lower chambers stained with crystal violet for 12 min, following fixation with 4% paraformaldehyde and quantification to evaluate HNSCC cell’s invasion capacity.

### circRNA pull-down and mass spectrometry

circRNA-binding proteins in HNSCC cells were characterized by circRNA pull-down assay using a Magnetic RNA-Protein Pull-Down Kit (Pierce, Thermo Fisher Scientific) as previously described with minor modifications [17]. Briefly, biotin-labeled probes (antisense-biotin: 5’-CCACCAACACCUGAUGUUCUGCAUGAAUaaa-3’-Biotin) targeting circ_0000045, or the negative control-biotin (NC-biotin: 5′-UUCUCCGAACGUGUCACGUdTdT-3′-Biotin), or sense-biotin (5’-AUUCAUGCAGAACAUCAGGUGUUGGUGGaaa-3’-Biotin) were incubated with RNA Capture Buffer for 30 min at room temperature, which was then mixed with streptavidin magnetic beads (80 μl), followed by two wash rounds with TRIS buffer, incubation for 25 min at room temperature, and two wash rounds with TRIS buffer again. Subsequently, these pre-treated magnetic beads labeled with probes were mixed with HNSCC cell lysates, after which they were subjected to overnight incubation at 4°C with gentle rotation. After washing twice with wash buffer, we conducted elution of circRNA-associated proteins by incubating the magnetic beads with 60 μl Elution Buffer at 37°C for 45 min. Proteins in the elute were then separated by SDS-PAGE and stained through silver staining, followed by a final identification by LC-MS/MS spectrometry as previously introduced [17]. Successively, functional categorization of RBPs was conducted using KEGG (Kyoto Encyclopedia of Genes and Genomes) (www.genome.jp/kegg/) [23], the enrichment of RBPs in molecular pathways was analyzed using GSEA (Gene Set Enrichment Analysis) (www.gsea-msigdb.org) [24], followed by a final prediction of binding between circRNA with RBPs, using the CSCD (cancer-specific circRNA database) platform (http://gb.whu.edu.cn/CSCD) [25].

### Western blotting

According to the manufacturer’s instructions, total proteins were extracted from HNSCC cells using the RIPA lysis and extraction buffer (#89900; Thermo Fishier Scientific). The protein concentration from HNSCC cells was then determined by the BCA method (#A53225; Thermo Fishier Scientific). Approximately 25-35 μg total proteins were mixed with a loading buffer and heated at 100°C for 5 min, followed by separation in 12% SDS-PAGE, blotting on PVDF membranes, subsequent blocking with 5% BSA solution at room temperature for 2 h-3 h, incubation with a specific primary antibody solution at 4°C overnight, incubation again with a secondary antibody solution for 2 h at room temperature, and a final development using Super Signal West Atto ECL substrates (#A38555; Thermo Fishier Scientific) according to the manufacturer’s instructions. Antibodies used in this study include; anti-JNK2 (dilution: 1:1000; #CST9258; CST), anti-P38 (dilution: 1:1000; #CST8690S; CST), anti-HSP70 (dilution: 1:2000; #AB182844; Abcam), anti-GAPDH (dilution: 1:1000; #SC25778; Santa Cruz), and Goat-anti-rabbit secondary antibody (dilution: 1:5000; #111-035-003; Jacson).

### Statistical analysis

The statistical analysis in this study was conducted using SPSS 20.0 to quantitative data in the form of mean ± SD (standard deviation), which was based on at least three biological replicates, after which assessment was conducted using Student’s T-test or ANOVA (analysis of variance) as appropriate. *P* < 0.05 was used as the threshold for significant differences.

## Results

### Identification of circRNAs differentially expressed in HNSCC tissue samples.

**Fig. 1:**
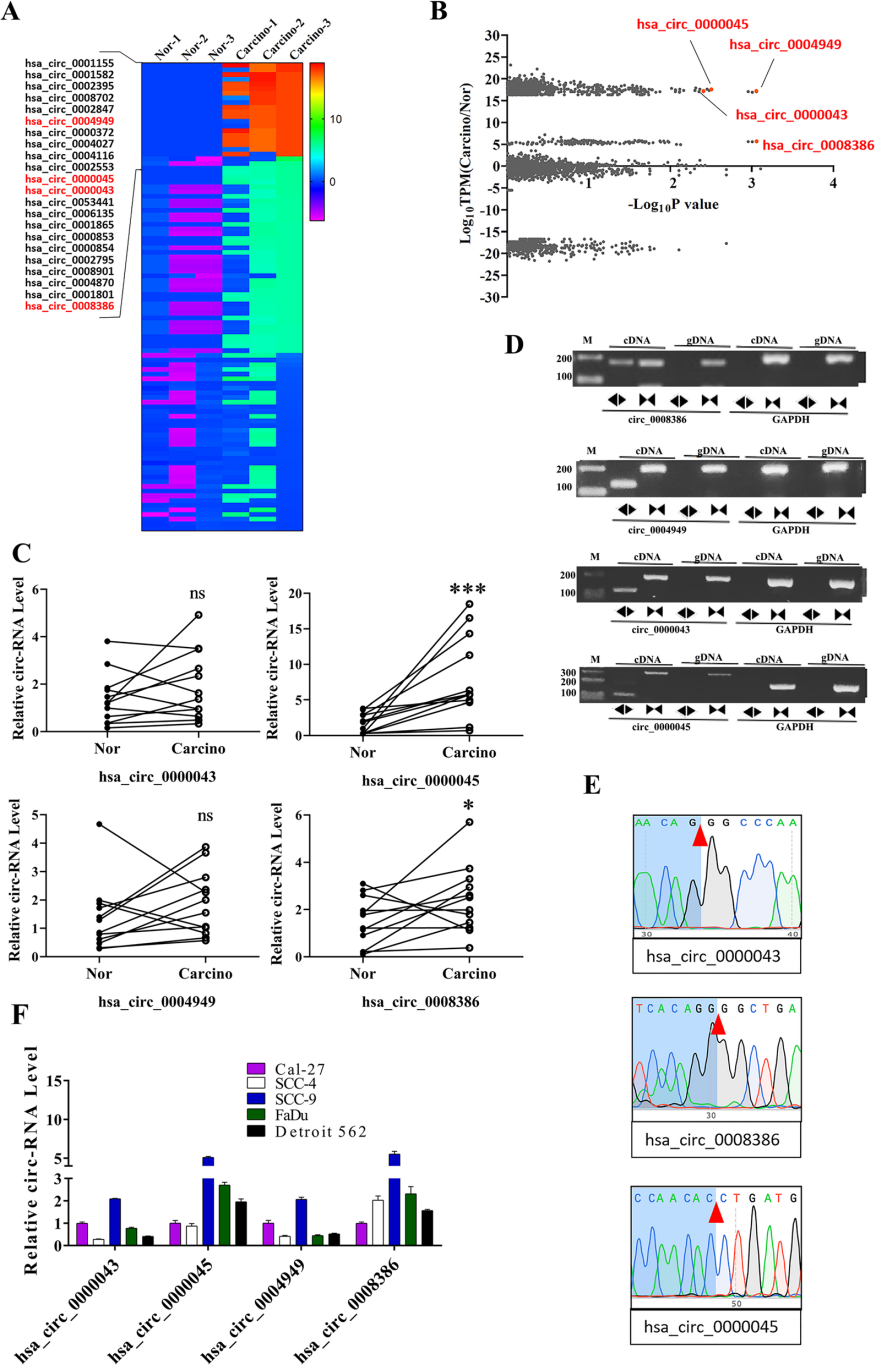
Differential expression of circRNAs in HNSCC tissues and cell lines. **A** A heatmap showing the differential expression of circRNAs between cancerous and adjacent non-cancerous tongue tissue samples, collected from three patients with HNSCC. RNA sequencing was conducted to characterize and quantify circRNA’s expression, after which the circRNA of interest having an elevated expression in HNSCC tissue samples were highlighted in red. **B** Volcano plots presenting the differential expression of circRNAs in HNSCC tissue samples. The Log_10_TPM (Carcino/Nor) and Log_10_P values are shown in the Y and X axes. When circRNA’s expression level is 0, we assumed 0 as 0.000000001 to calculate the value of Log10TPM. **C** Relative expressional levels of four representative circRNAs in HNSCC tissues. Levels of circRNA expression in HNSCC tissues were assessed using quantitative RT-PCR. N=12 (patient 3-14). **D** RT-PCR-based validation of circRNA synthesis in HNSCC tissue samples (from patient 3). The identities of circ_0008386, circ_0004949, circ_0000043, and circ_0000045 were confirmed by PCR using divergent primers, genomic DNA, or convergent primers as negative controls. GAPDH was a positive control. **E** Identification of circRNA junction sites through DNA Sanger sequencing. PCR products from panel D were used for DNA sequencing, after which junction sites were indicated using red triangles. **F** Relative expressional levels of four representative circRNAs in HNSCC cell lines. Levels of circRNA expression in HNSCC cell lines were assessed using quantitative RT-PCR. *N* = 3; Nor: adjacent normal tissues; Carcino: HNSCC tissues; cDNA: complementary DNA; gDNA: genomic DNA; GAPDH: glyceraldehyde-3-phosphate dehydrogenase

To explore the possible involvement of circRNAs during HNSCC’s pathogenesis, we conducted an RNA sequencing characterization of circRNAs showing significantly differential expression between cancerous and adjacent tongue tissue samples obtained from three oral tongue squamous cell carcinoma patients (Fig. 1A and B). We observed that the expression of 22 circRNAs in these HNSCC tissue samples was significantly higher than the corresponding adjacent non-cancerous normal tissues, such as circ_0000045, circ_0004949, circ_0000043, and circ_0008386 (Fig. 1A and B, Supplemental Table 2). Moreover, we detected the expression of circ_0000045, circ_0004949, circ_0000043, and circ_0008386 in HNSCC tissue samples and adjacent non-cancerous normal tissues (N=12, from patient 3-14, patient 1 and patient 2 samples were used up for sequencing), and the results showed that only the expression of circ_0000045 and circ_0008386 in HNSCC tissue samples was significantly higher than the corresponding adjacent non-cancerous normal tissues (Fig. 1C). Subsequently, the circular structure identities of these four differentially expressed circRNAs in HNSCC tissue samples from pateint 3 were further validated by RT-PCR, convergent primers targeting circular RNAs were used as convergent primers targeting the linear RNA molecules control (Fig. 1D). Furthermore, junction sites of circ_0000045, circ_0000043, and circ_0008386 were further verified by DNA Sanger’s sequencing using corresponding PCR products (Fig. 1E). Afterward, we analyzed the relative expression levels of the four circRNAs mentioned above in several HNSCC cell lines by qRT-PCR. We observed that these four circRNAs showed significantly high expression in FaDu and SCC-9 cells (Fig. 1F). Among them, the expression of hsa_circ_0000045 in FaDu and SCC-9 cells was much higher than that of the other three cell lines (Fig. 1F). Therefore, FaDu and SCC-9 cells were selected to analyze circRNA functions in HNSCC cellular processes further.

### circ_0000045 knockdown repressed HNSCC cell proliferation, migration, and invasion.

**Fig. 2:**
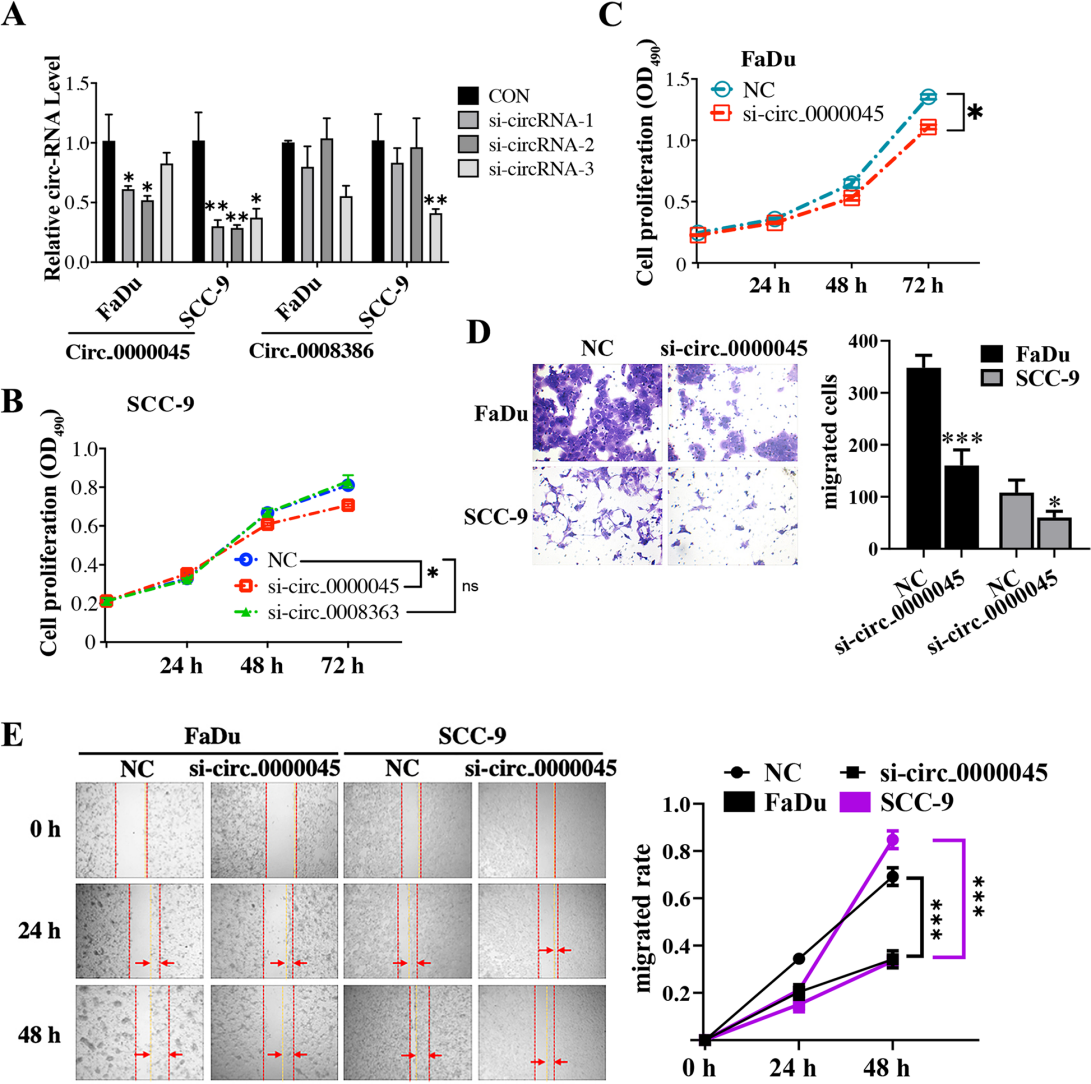
HNSCC cell proliferation, migration, and invasion inhibition through circ_0000045 silencing. **A** The relative expression of circ_0000045 and circ_0008386 in FaDu and SCC-9 cells after siRNA transfection. Expression of circ_0000045 was measured through qRT-PCR. **B** Alterations in SCC cell proliferation rates induced by silencing circ_0000045 and circ_0008386. **C** The decrease in FaDu cell proliferation rates caused by silencing circ_0000045’s expression. MTS method was performed to assess the proliferation rates of both FaDu and SCC-9 cells. **D** Inhibition of FaDu and SCC-9 cell invasion capacities caused by silenced circ_0000045 expression. Cell invasion was assessed using the Transwell system. **E** Changes in migration rates of FaDu and SCC-9 cells induced by the knockdown of circ_0000045’s expression. A wound-healing assay was used to evaluate the migration speeds of HNSCC cells. N = 3; CON: control; si: siRNA; NC: negative control of siRNA; OD490: absorbance at 490 nm; *P < 0.05; **P < 0.01

To investigate potential functions of circRNAs during HNSCC’s development, we silenced the expression of circ_0000045 and circ_0008386 in two HNSCC cell lines by transfecting with specific siRNAs (Fig. 2A). Compared with the control group, siRNA transfections significantly reduced the expression of circ_0000045 in both FaDu and SCC-9 cells, while the expression of circ_0008386 was successfully silenced only in SCC-9 cells (Fig. 2A). Furthermore, through MTS, we showed that the proliferation rates of SCC-9 cells were repressed by circ_0000045 knockdown, other than circ_0008386 knockdown (Fig. 2B). Similarly, knocking down circ_0000045 substantially suppressed cell proliferation rates in FaDu cells (Fig. 2C). Therefore, further functional analysis of circ_0000045 was conducted in FaDu and SCC-9 cells. We also observed using the Transwell system that circ_0000045’s knockdown impaired the invasion capacities of both FaDu and SCC-9 cells, compared with the NC groups (Fig. 2D). Additionally, our wound-healing assay showed that the migration capacities of both FaDu and SCC-9 cells were repressed by silencing circ_0000045’s expression, compared with negative controls (Fig. 2E). Therefore, these results indicated the significant roles of circ_0000045 in promoting HNSCC cell proliferation, migration, and invasion.

### circ_0000045’s overexpression promoted HNSCC cell proliferation, migration, and invasion.

**Fig. 3:**
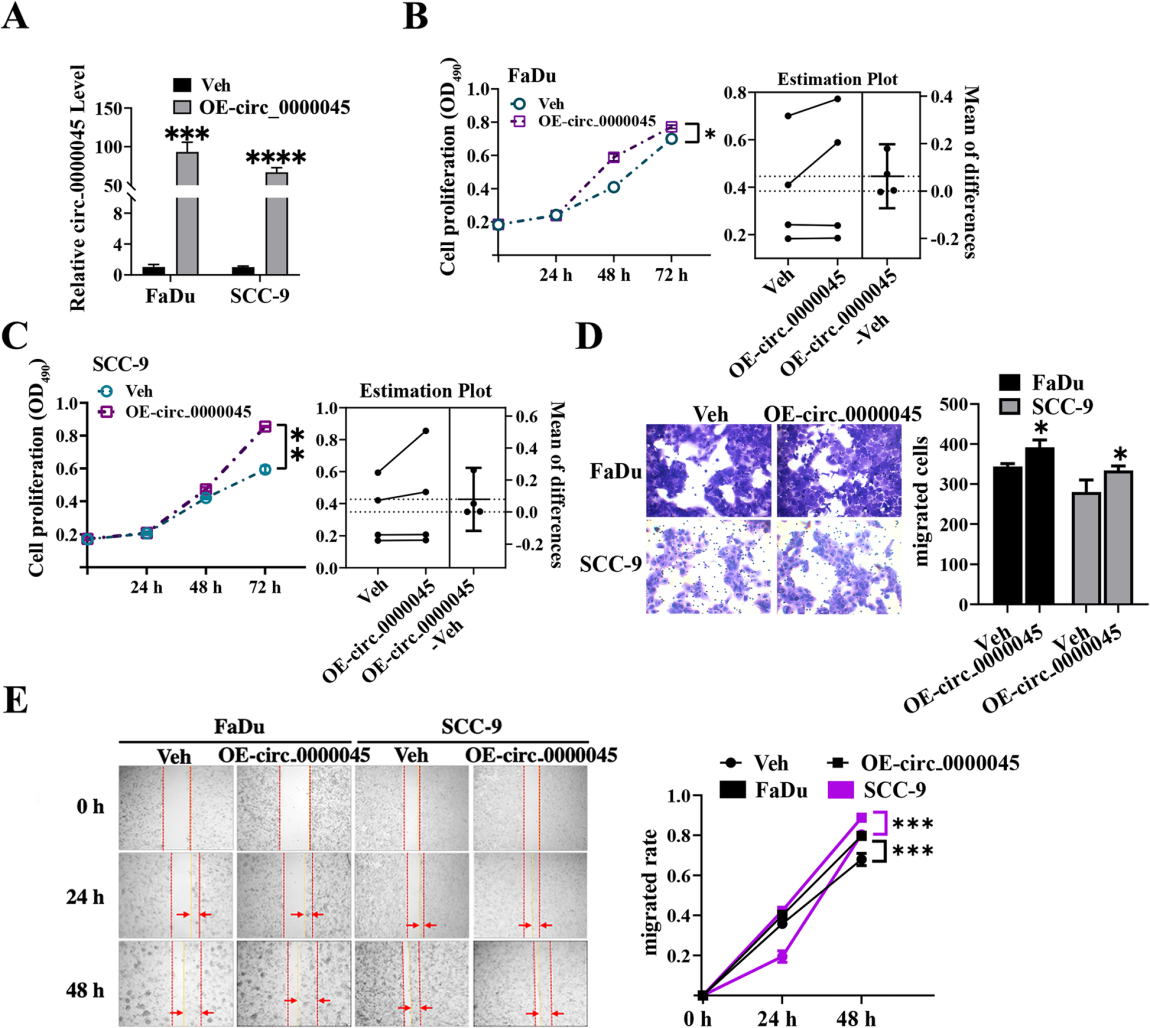
Promotion of HNSCC cell proliferation, migration, and invasion through circ_0000045’s overexpression. **A** Great increase in circ_0000045’s expression in FaDu and SCC-9 cells caused by transfection with overexpressing vectors. The expression of circ_0000045 in HNSCC cells was detected through quantitative RT-PCR using cells transfected with empty vectors as negative controls. **B**, **C** Significant elevation in FaDu and SCC-9 cell proliferation rates induced through the overexpression of circ_0000045. The proliferation of FaDu (**B**) and SCC-9 (**C**) cells was determined by MTS. **D** Enhanced invasion capacities of FaDu and SCC-9 cells due to circ_0000045’s overexpression. The invasion of HNSCC cells was assessed through the Transwell system. **E** High migration speeds of FaDu and SCC-9 cells induced by the overexpression of circ_0000045. The wound-healing assay was conducted to evaluate the migration of HNSCC cells. N = 3; Veh: vector; OE: overexpression; OD490: absorbance at 490 nm; *P < 0.05; **P < 0.01; ***P < 0.001

To further validate the cellular functions of circ_0000045, we overexpressed circ_0000045 in both FaDu and SCC-9 cells (Fig. 3A). Through quantitative RT-PCR, we confirmed that the relative expression of circ_0000045 in both FaDu and SCC-9 cells was significantly increased by transfection with overexpressing vectors, which was in contrast to that observed in empty vector groups (Fig. 3A). Subsequently, MTS revealed that the proliferation rates of both FaDu and SCC-9 cells were elevated by circ_0000045’s overexpression, compared with the vector control groups (Fig. 3B and C). Moreover, using the Transwell system, we observed that the invasion capacities of both FaDu and SCC-9 cells were enhanced by the overexpression of circ_0000045, compared with the empty vector control groups (Fig. 3D). Furthermore, we showed through the wound-healing assay that the migration speeds of both FaDu and SCC-9 cells were also elevated by the overexpression of circ_0000045, compared with the control groups (Fig. 3E). Therefore, these results confirmed the essential roles of circ_0000045 in promoting HNSCC cell proliferation, migration, and invasion capacities.

### circ_0000045 was associated with multiple RBPs in HNSCC cells.

**Fig. 4:**
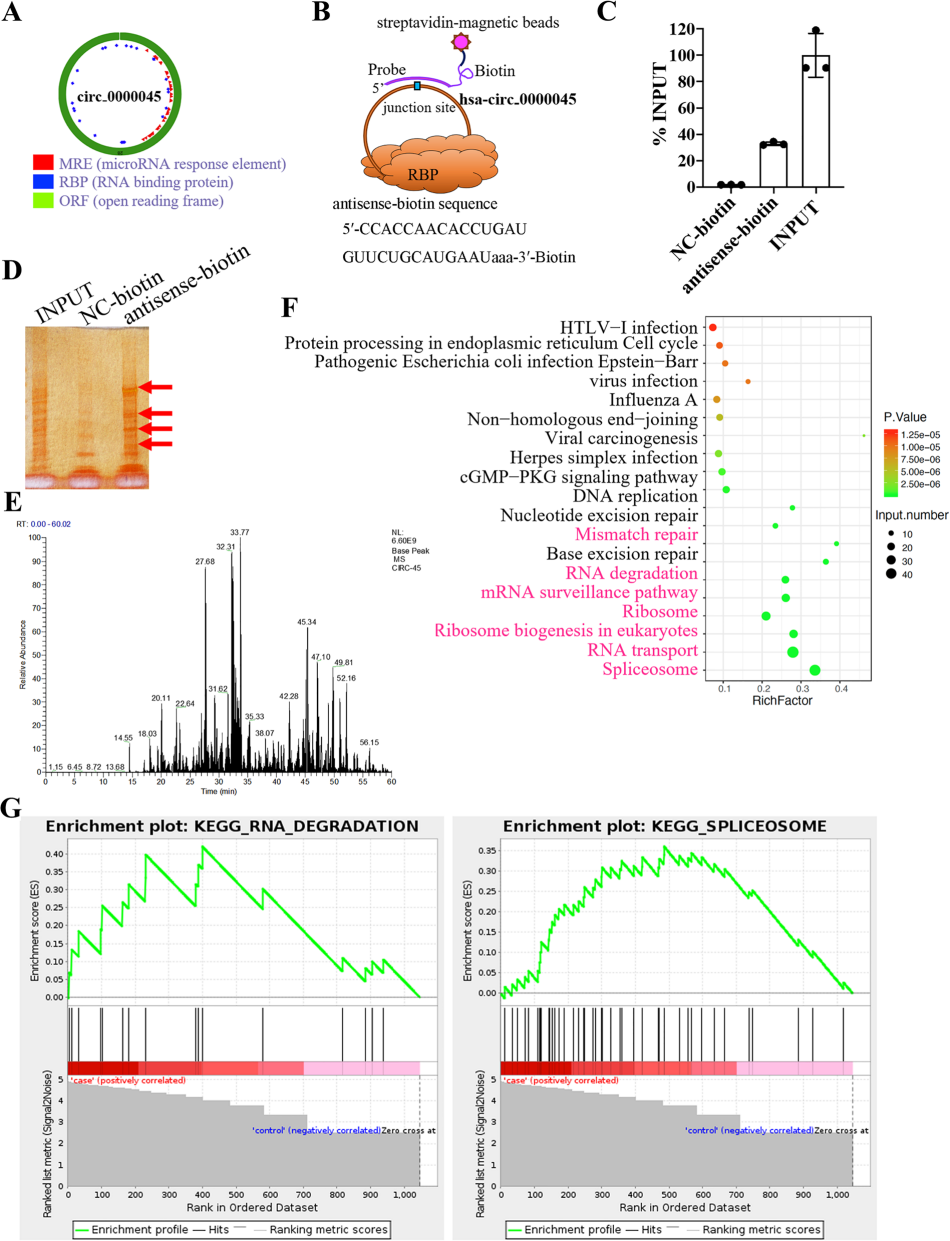
The identification of circ_0000045-binding RBPs in HNSCC cells. **A** The predicted association of circ_0000045 with multiple RPBs in HNSCC cells. The potential association of circ_0000045 with RBPs was predicted using the CSCD database (http://gb.whu.edu.cn/CSCD). **B** A schematic presentation of the circRNA pull-down assay used to purify circ_0000045-associated RBPs in HNSCC cells. **C** The relative quantitation of proteins eluted with biotin-labeled probes targeting circ_0000045 in SCC-9 cells using a circRNA pull-down, *N* = 3. **D** Silver staining of circ_0000045-binding RBPs eluted with biotin-labeled probes in SCC-9 cells during circRNA pull-down. **E** A mass spectrometric plot, showing the identification of RBPs associated with circ_0000045 in SCC-9 cells. **F** Functional categorization of circ_0000045-binding RBPs in SCC-9 cells. The enrichment of RBPs in KEGG pathways was conducted using the DAVID system. **G** Significant enrichment of circ_0000045-binding RBPs from SCC-9 cells in RNA degradation and spliceosome pathways. Investigation of enriched molecular pathways was conducted using the GSEA software. NC-biotin: negative control of biotin-labeled probes targeting circ_0000045; KEGG: Kyoto Encyclopedia of Genes and Genomes; *P < 0.05; **P < 0.01

Based on bioinformatic analysis (gb.whu.edu.cn/CSCD), circ_0000045 was predicted to bind with several RNA-binding proteins (RBPs) (Fig. 4A). Therefore, to further investigate molecular mechanisms underlying circ_0000045-regulated HNSCC cell functions, we explored the potential involvement of RBPs in these processes. In Fig. 4B, we showed how the circRNA pull-down assay was used to purify circ_0000045-associated RBPs in HNSCC cells. In the next experiment, we quantified RBP proteins eluted during the circRNA pull-down assay to show that the antisense-biotin group obtained significantly elevated protein contents, compared with the NC-biotin group in SCC-9 cells (Fig. 4C). To further verified this phenomenon, we performed circRNA pull-down assay in SCC-4 and SCC-4 OE-circ_0000045 cells, and the results indicated that antisense-biotin group obtained significantly elevated protein contents, compared with the NC-biotin group and sense-biotin group (Supplemental Figure 1). We further separated these eluted proteins in SCC-9 cells by SDS-PAGE, followed by silver staining, which disclosed multiple protein bands that were proposed as circ_0000045-binding proteins (Fig. 4D). Subsequently, these RBP protein bands were characterized by LC-MS/MS (Fig. 4E), which identified that a large group of RBPs was associated with circ_0000045 in HNSCC cells (Supplemental Table 3). Our functional categorization also showed that these RBPs were enriched in multiple biological processes, including RNA degradation, spliceosome, mismatch repair, mRNA surveillance pathway, RNA transport, and ribosome biogenesis (Fig. 4F). Moreover, through GSEA analysis, we further highlighted the significant enrichment of circ_0000045-binding RBPs in RNA degradation and spliceosome pathways, such as the HSPA8 (heat shock 70kDa protein 8) pathway, which belongs to the HSP70 (heat shock 70kDa protein) family (Fig. 4G; Supplemental Table 3). Thus, these results maybe indicated that circ_0000045 could regulate HNSCC cell functions through RBP binding to modulate the expression of target genes.

### circ_0000045 knockdown elevated HSP70 to suppress MAPK signaling in HNSCC cells.

**Fig. 5:**
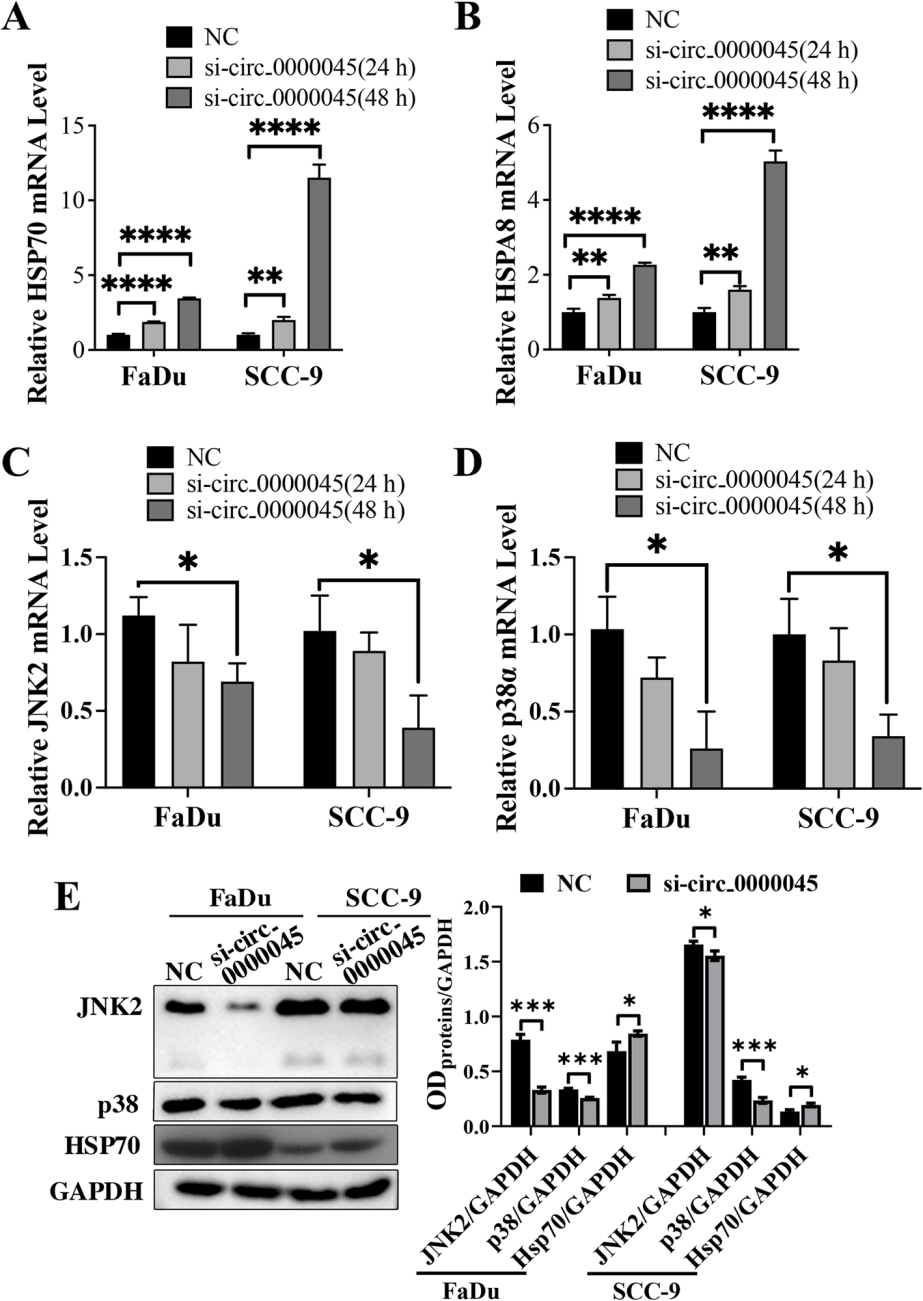
Modulation of HSP70 and MAPK signaling by circ_0000045 silencing in HNSCC cells. **A**, **B** The elevation of HSP70 and HSPA8 gene expression in HNSCC cells by circ_0000045 silencing. The expression of HSP70 (**A**) and HSPA8 (**B**) genes were determined in both FaDu and SCC-9 cells by quantitative RT-PCR. **C**, **D** Significant decrease in JNK2 and p38 gene mRNA levels in both FaDu and SCC-9 cells caused by circ_0000045 silencing. Quantitative RT-PCR was conducted to analyze JNK2 and P38 mRNA levels. **E** Alterations in HSP70, JNK2, and P38 protein levels in FaDu and SCC-9 cells with silenced circ_0000045 expression at 48 h. Western blotting was used to measure protein abundances in HNSCC cells. However, GAPDH was detected as the internal standard. *N* = 3; HSP70: heat shock 70kDa protein; HSPA8: heat shock 70kDa protein 8; NC: negative control of siRNA; JNK2: c-Jun N-terminal kinase 2; GAPDH: glyceraldehyde-3-phosphate dehydrogenase; *P < 0.05; **P < 0.01

To further confirm the association of HSP70 with downstream signaling pathways, we detected the expression of HSP70 and HSPA8 proteins first, including JNK2 (c-Jun N-terminal kinase 2) and P38 expression in FaDu and SCC-9 cells transfected with siRNA targeting circ_0000045. Then, through quantitative RT-PCR, we showed that the expression of HSP70 and HSPA8 mRNA levels in both FaDu and SCC-9 cells were significantly elevated by circ_0000045 silencing, especially at 48 h after the siRNA transfection, which was in contrast to the NC groups (Fig. 5A and B). Alternatively, we observed that mRNA levels of JNK2 and p38 genes in both FaDu and SCC-9 cells were effectively reduced by silencing circ_0000045 expression, compared with the NC group (Fig. 5C and D). Similarly, subsequent Western blotting also revealed an increase in the HSP70 protein, including a substantial decrease in JNK2 and p38 proteins in both FaDu and SCC-9 cells with silenced circ_0000045 expression at 48 h (Fig. 5E). To verify whether the effects of circ_0000045 on JNK and p38 is blocked without Hsp70, si-HSP70 and si-circ_0000045 were transfected into FaDu and SCC-9 cells for 48 h. Then, cells samples were collected for Western blotting. Results showed that silence of Hsp70 enhanced the expression of JNK2 and P38 protein, compared with NC group (Supplemental Figure 2). Moreover, knockdown of Hsp70 along with circ_0000045 siRNA decrease the exrpession of JNK2 and P38 protein, compared with si-HSP70 group, although there was no statistical difference for JNK2 protein expression in SCC-9 cells (Supplemental Figure 2). Hence, these results showed the roles of circ_0000045 in suppressing HSP70’s expression and promoting MAPK signaling in HNSCC cells.

### circ_0000045 suppressed HSP70 to enhance MAPK signaling in HNSCC cells.

**Fig. 6:**
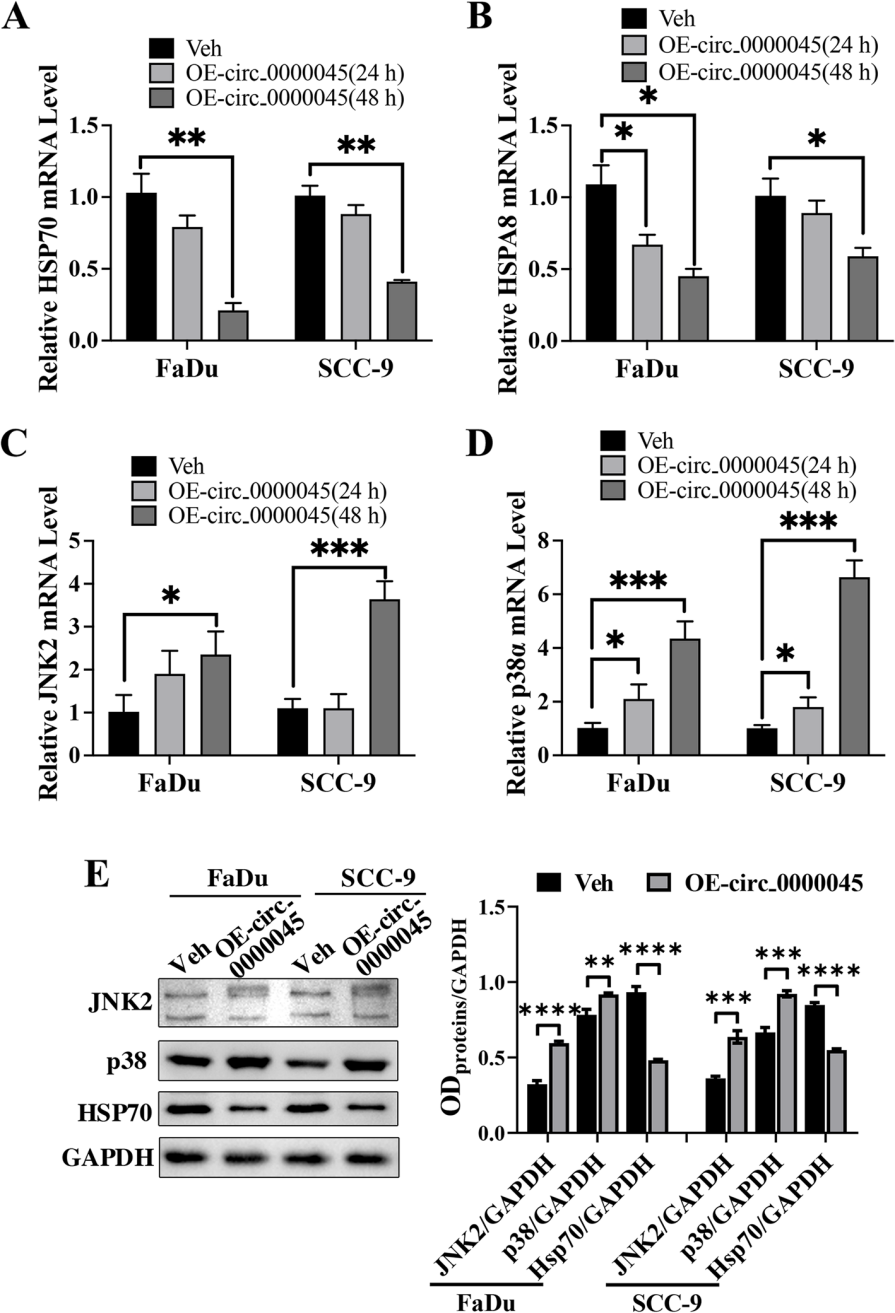
Alterations in HSP70’s expression and MAPK signaling by circ_0000045 overexpression in HNSCC cells. **A**, **B** Decrease in expression of HSP70 and HSPA8 genes in HNSCC cells caused by circ_0000045’s overexpression. Levels of HSP70 (**A**) and HSPA8 (**B**) mRNAs in HNSCC cells were measured by quantitative RT-PCR. **C**, **D** Elevations in JNK2 and p38 mRNA levels in FaDu and SCC-9 cells with circ_0000045’s overexpression. Quantitative RT-PCR was conducted to measure JNK2 and P38 mRNA contents in HNSCC cells. **E** Changes in HSP70, JNK2, and P38 protein abundances in HNSCC cells with elevated circ_0000045’s expression. Western blotting was conducted to detect protein levels in HNSCC cells after transfection for 48 h. However, GAPDH was used as the internal standard. *N* = 3; Veh: vector; OE: overexpression; HSP70: heat shock 70kDa protein; HSPA8: heat shock 70kDa protein 8; JNK2: c-Jun N-terminal kinase 2; GAPDH: glyceraldehyde-3-phosphate dehydrogenase; *P < 0.05; **P < 0.01

To further validate the effects of circ_0000045 in regulating HSP70’s expression and MAPK signaling, we first detected the expression of HSP70 proteins and MAPK signaling components in HNSCC cells overexpressing circ_0000045. Then, as expected, we showed by quantitative RT-PCR that the mRNA levels of both HSP70 and HSPA8 in FaDu and SCC-9 cells were remarkably down-regulated by the overexpression of circ_0000045, compared with the control groups (Fig. 6A and B). Contrarily, we also demonstrated by quantitative RT-PCR that compared with the control groups, mRNA levels of JNK2 and P38 genes in FaDu and SCC-9 cells were significantly increased by circ_0000045’s overexpression (Fig. 6C and D). Finally, Western blotting also detected a reduction in HSP70 proteins and an elevation of JNK2 and P38 proteins in these two HNSCC cell lines induced by circ_0000045 overexpression at 48 h (Fig. 6E). Together, these assays confirmed that circ_0000045 repressed HSP70 to promote MAPK signaling in HNSCC cells.

## Discussion

The development of multiple human cancers is essentially mediated by complex signaling networks regulated by various non-coding RNAs, such as circular RNAs (circRNAs) [9]. However, the specific roles of circRNAs and their downstream molecular mechanisms in HNSCC pathogenesis remains poorly understood. Therefore, we investigated the roles of circRNAs in HNSCC’s pathogenic processes in this study by conducting a large-scale characterization of circRNAs differentially expressed in HNSCC tissue samples through RNA sequencing. Among them, specific roles of circ_0000045 in regulating HNSCC cell proliferation, migration, and invasion were further assessed via silencing or overexpressing circ_0000045 in FaDu and SCC-9 cells. Results displayed for the first time the essential functions of circ_0000045 in enhancing HNSCC cell proliferation, migration, and invasion. Next, for the underlying mechanisms, we conducted a proteomic analysis of circ_0000045 associated with RBPs, following circRNA pull down. As observed, circ_0000045 bound with multiple proteins related to RNA degradation and spliceosome in HNSCC cells, such as the HSP70 protein family. Finally, we further revealed that circ_0000045 suppressed HSP70’s expression and promoted the MAPK signaling cascade in HNSCC cells. These transcriptome and proteomic findings revealed novel molecular mechanisms of HNSCC’s pathogenesis based on non-coding RNAs and RBPs.

As introduced above, circRNAs act as potent regulators of cellular signaling pathways via modulating various cellular processes, including cell viability, proliferation, migration, and invasion, due to their significant alterations of functional gene expression or their association with microRNA and proteins [9, 26, 27]. During the past decades, the significance of circRNA in pathogenic processes has prevalently been reported in various human disorders, such as cardiovascular diseases, neurodegeneration, and malignant diseases [28–31]. Previous research also displayed the widespread association of circRNA differential expression in HNSCC’s development, progression, metastasis, prognosis, and sensitivity to chemotherapy [11–14, 32]. However, compared with the significant alteration of circRNA profiles in HNSCC tissues and cell lines, our recent knowledge of circRNA-regulated HNSCC pathogenic mechanism is limited. Here, we identified significant changes in circRNA’s expression in cancerous tissues collected from patients with oral tongue squamous cell carcinoma. Further investigations showed the influences of circ_0000045 in the proliferation, migration, and invasion capacities of HNSCC cell lines. The effective regulation of HNSCC cell functions by circ_0000045 further stressed the essential pathogenic roles of circRNAs in HNSCC development. As observed, the prevalent regulation of HNSCC cell proliferation, migration, and invasion suggested that circRNAs were involved in differential cellular processes and pathogenic stages during HNSCC’s progression.

Recent extensive research has also demonstrated that HNSCC’s pathogenesis was regulated by multiple RBPs [19, 33]. Functional gene expression networks controlled by RBPs, such as ADAR1, DDX3, FXR1, and ESRP1/2 (epithelial splicing regulatory proteins 1/2), were closely associated with HNSCC cell’s migration and invasiveness, including cancer metastasis and poor prognosis [19], thereby serving as new promising targets for HNSCC’s treatment. Interestingly, although RBP ESRP1/2 could bind with several circRNAs, such as circUHRF1 in HNSCC cells, ESRP1 enhanced the biogenesis of circUHRF1 via a direct association to regulate c-MYC signaling and HNSCC development [34], which was indicative of the interaction between RBPs and circRNAs in HNSCC’s pathogenesis. Furthermore, recent progress showed that RBPs and circRNAs possess reciprocal interplay in various processes [16]. However, the regulation of RPBs by circRNAs in HNSCC cells has not been reported previously. We clearly showed that circ_0000045 bound to a group of RBPs in HNSCC cells, further highlighting the significance of circRNA-RBP interaction in cancer development. More importantly, we proved here that although the expression of one RBP, the HSP70 family member; HSPA8, was substantially repressed by circ_0000045 in HNSCC cells at the mRNA level (WB results from HSPA8 were not well exhibited after we used different primary antibodies). Our discoveries, therefore, confirmed the existence of a reciprocal interaction between circRNA and RBP in HNSCC development. In light of the known roles of HSP70 in HNSCC carcinogenesis [35–37], our discovery of circRNA-regulated HSP70 expression revealed a new pathway accounting for HNSCC’s molecular pathogenesis.

Mitogen-activated protein kinases (MAPKs)-associated signaling cascades play essential roles in cancer development and progression [38]. As observed, the ERK1/2 (Extracellular Signal Regulated Kinase 1/2), which mitogens can activate, has been established as a significant oncogenic signaling pathway in cancer cells, including HNSCC development [39, 40]. Moreover, besides ERK MAPKs, JNK (Jun N-terminal kinase) and P38 serve as two other critical MAPK signaling pathways also closely associated with cancer development, which can be activated by environmental stresses to regulate cell proliferation and migration [38]. For instance, the activation of JNK1/2 and ERK1/2 signaling cascades were recently shown to be significantly correlated with the differentiation of HNSCC tumors [41, 42]. However, molecular mechanisms underlying the alteration of MAPK signaling during HNSCC development remains poorly understood. Here, we proved that the circRNA effectively promoted JNK2 and P38 proteins' expression, circ_0000045, which disclosed a circRNA-regulated MAPK signaling pathway in HNSCC cells. Alternatively, previous reports observed that HSP70 modulated the MAPK signaling in various physiological and pathogenic contexts [43, 44]. Likewise the correlated alteration of HSP70 and JNK2/P38’s expression shown in this study in HNSCC cells supported the interaction of HSP70 and MAPK signaling in cancer development. Nevertheless, whether circ_0000045 regulated MAPK signaling via HSP70 needs more studies to verify. More studies should also be conducted to investigate whether circ_0000045 directly (or indirectly) binds HSP70, which plays a major role in regulating Hsp70, including whether circ_0000045 binds only HSP70, or many other proteins.

In summary, we reported in this study that circ_0000045, whose expression was significantly elevated in HNSCC tissues, promoted the proliferation, migration, and invasion of HNSCC cells maybe by associating with RBPs, such as HSP70, regulating JNK and P38 MAPK signaling pathways. Therefore, these results disclosed new molecular mechanisms driving HNSCC’s development and progression, which can also act as new targets for cancer diagnosis and treatment.

## Supplementary Information

**Additional file 1.**

**Additional file 2.**

**Additional file 3.**

**Additional file 4.**

**Additional file 5.**

**Additional file 6.**

## Data Availability

All data generated or analyzed during this study are included in this published article and the RNA sequencing datasets are available in [NCBI]: GEO accession number is GSE197138.

## Abbreviations

HNSCC: Head and neck squamous cell carcinomas
circRNAs: circular RNAs
EGFR: epidermal growth factor receptor
ncRNAs: Non-coding RNAs
SIRT3: Silent information regulator 3
mTOR: mammalian target of Rapamycin
MAPK: mitogen-activated protein kinase
RBPs: RNA-binding proteins
RBDs: RNA-binding domains
PDAC: pancreatic ductal adenocarcinoma
YBX1: Y-box binding protein 1
hnRNPK: heterogeneous nuclear ribonucleoprotein K
ADAR1: adenosine deaminase acting on RNA 1
DDX3: DEAD-box RNA helicase 3
TERC: Telomerase RNA Component
GSEA: Gene Set Enrichment Analysis
CSCD: cancer-specific circRNA database
SD: standard deviation
ANOVA: analysis of variance
JNK2: c-Jun N-terminal kinase 2
ESRP1/2: epithelial splicing regulatory proteins 1/2
JNK: Jun N-terminal kinase
