# Supplementary material for: circ_0000045 promotes proliferation, migration, and invasion of head and neck squamous cell carcinomas via regulating HSP70 and MAPK pathway
Source: BMC Cancer. 2022 Jul 20;22:799. doi: 10.1186/s12885-022-09880-y (PMC9297571; doi:10.1186/s12885-022-09880-y)
Supplement: Supplementary file 1 — Additional file 1. [file 12885_2022_9880_MOESM1_ESM.docx]

Supplemental table 1. The three patient information used in RNA sequencing.

| Patient number | Age (Years) | Gender | TNM staging |
| --- | --- | --- | --- |
| Patient 1 | 63 | Male | T3N2M0 |
| Patient 2 | 54 | Male | T3N1M0 |
| Patient 3 | 52 | Female | T2N0M0 |
| Patient 4 | 33 | Male | T3N0M0 |
| Patient 5 | 43 | Female | T3N2M0 |
| Patient 6 | 48 | Female | T2N2M0 |
| Patient 7 | 69 | Female | T2N0M0 |
| Patient 8 | 46 | Male | T2N0M0 |
| Patient 9 | 68 | Female | T2N0M0 |
| Patient 10 | 70 | Female | T2N0M0 |
| Patient 11 | 52 | Female | T4N2M0 |
| Patient 12 | 44 | Male | T2N0M0 |
| Patient 13 | 65 | Female | T4N0M0 |
| Patient 14 | 70 | Female | T2N0M0 |
