# Supplementary material for: circ_0000045 promotes proliferation, migration, and invasion of head and neck squamous cell carcinomas via regulating HSP70 and MAPK pathway
Source: BMC Cancer. 2022 Jul 20;22:799. doi: 10.1186/s12885-022-09880-y (PMC9297571; doi:10.1186/s12885-022-09880-y)
Supplement: Supplementary file 2 — Additional file 2. [file 12885_2022_9880_MOESM2_ESM.docx]

Supplemental table 2. The log_10_TPM (Carcino/Nor) value of 22 differential expressed circRNAs between cancerous and adjacent non-cancerous tongue tissues in Figure 1A showed in the form of heatmap. The right colour column was the value scale for determining colour, the pink indicated log_10_TPM (Carcino/Nor) value was lower than zero, and the blue indicated log_10_TPM (Carcino/Nor) value was equal to zero, the other colours indicated log_10_TPM (Carcino/Nor) value was more than zero.
