# Supplementary material for: circ_0000045 promotes proliferation, migration, and invasion of head and neck squamous cell carcinomas via regulating HSP70 and MAPK pathway
Source: BMC Cancer. 2022 Jul 20;22:799. doi: 10.1186/s12885-022-09880-y (PMC9297571; doi:10.1186/s12885-022-09880-y)
Supplement: Supplementary file 3 — Additional file 3. [file 12885_2022_9880_MOESM3_ESM.docx]

| **Supplemental table 3. Proteins indentified by circRNA pull down assay.** | |
| --- | --- |
| **Enrichment plot: KEGG_RNA_DEGRADATION** | |
| TTC37 | tetratricopeptide Repeat Domain 37 |
| SKIV2L | superkiller viralicidic activity 2-like (S. cerevisiae) |
| EXOSC10 | exosome component 10 |
| WDR61 | WD repeat domain 61 |
| HSPD1 | heat shock 60kDa protein 1 (chaperonin) |
| EXOSC3 | exosome component 3 |
| EXOSC9 | exosome component 9 |
| EXOSC8 | exosome component 8 |
| EXOSC2 | exosome component 2 |
| EXOSC6 | exosome component 6 |
| HSPA9 | heat Shock Protein Family A (Hsp70) Member 9 |
| ENO1 | enolase 1. (alpha) |
| DIS3 | DIS3 Homolog, exosome dndoribonuclease and 3'-5' exoribonuclease |
| DDX6 | DEAD (Asp-Glu-Ala-Asp) box polypeptide 6 |
| LSM4 | LSM4 homolog. U6 small nuclear RNA associated (S. cerevisiae) |
| EXOSC4 | exosome component 4 |
| XRN1 | 5'-3' exoribonuclease 1 |
|  |  |
| **Enrichment plot: KEGG_SPLICEOSOME** | |
| HNRNPM | heterogeneous nuclear ribonucleoprotein M |
| EFTUD2 | elongation factor Tu GTP binding domain containing 2 |
| HSPA1B | heat shock 70kDa protein 1B |
| DDX5 | DEAD (Asp-Glu-Ala-Asp) box polypeptide 5 |
| SF3A1 | splicing factor 3a, subunit 1, 120kDa |
| EIF4A3 | eukaryotic translation initiation factor 4A3 |
| HSPA8 | heat shock 70kDa protein 8 |
| RBM25 | RNA binding motif protein 25 |
| SE3B2 | splicing factor 3b, subunit 2, 145kDa |
| SNW1 | SNW domain containing 1 |
| HNRNPU | heterogeneous nuclear ribonucleoprotein U |
| SART1 | squamous cell carcinoma antigen recognised by T cells |
| PUF60 | poly(U) binding splicing factor 60 |
| PLRG1 | pleiotropic regulator 1 (prl1 homolog, Arabidopsis) |
| U2AF2 | U2 small nuclear RNA auxiliary factor 2 |
| SRSF7 | serine and arginine rich splicing factor 7 |
| CHERP | calcium homeostasis endoplasmic reticulum protein |
| SRSF1 | serine and arginine rich splicing factor 1 |
| DHX15 | DEAH (Asp-Glu-Ala-His) box polypeptide 15 |
| RBMX | RNA binding motif protein, X-linked |
| U2AF1 | U2 small nuclear RNA auxiliary factor 1 |
| SF3B1 | splicing factor 3b, subunit 1, 155kDa |
| USP39 | ubiquitin specific peptidase 39 |
| PRPF40A | PRP40 pre-mRNA processing factor 40 homolog A (yeast) |
| CINNBL1 | catenin, beta like 1 |
| PRPF6 | PRP6 pre-mRNA processing factor 6 homolog (S. cerevisiae) |
| ACIN1 | apoptotic chromatin condensation inducer 1 |
| SNRNP200 | small nuclear ribonucleoprotein U5 subunit 200 |
| PRPF8 | PRP8 pre-mRNA processing factor 8 homolog (S. cerevisiae) |
| SRSF10 | serine and arginine rich splicing factor 10 |
| PRPF19 | PRP19/PSO4 pre-mRNA processing factor 19 homolog (S. cerevisiae) |
| U2SURP | U2 SnRNP associated SURP domain containing |
| DHX16 | DEAH (Asp-Glu-Ala-His) box polypeptide 16 |
| DDX42 | DEAD (Asp-Glu-Ala-Asp) box polypeptide 42 |
| PRPF31 | PRP31 pre-mRNA processing factor 31 homolog (S. cerevisiae) |
| RBM17 | RNA binding motif protein 17 |
| SF3A2 | splicing factor 3a, subunit 2, 66kDa |
| PCBP1 | poly(rC) binding protein 1 |
| TCERG1 | transcription elongation regulator 1 |
| CWC15 | CWC15 spliceosome associated protein homolog |
| THOC2 | THO complex 2 |
| LSM4 | LSM4 homolog. U6 small nuclear RNA associated (S. cerevisiae) |
| DDX23 | DEAD (Asp-Glu-Ala-Asp) box polypeptide 23 |
| SNRPB | small nuclear ribonucleoprotein polypeptides B and B1 |
