# Supplementary figures and images for: circ_0000045 promotes proliferation, migration, and invasion of head and neck squamous cell carcinomas via regulating HSP70 and MAPK pathway
Source: BMC Cancer. 2022 Jul 20;22:799. doi: 10.1186/s12885-022-09880-y (PMC9297571; doi:10.1186/s12885-022-09880-y)

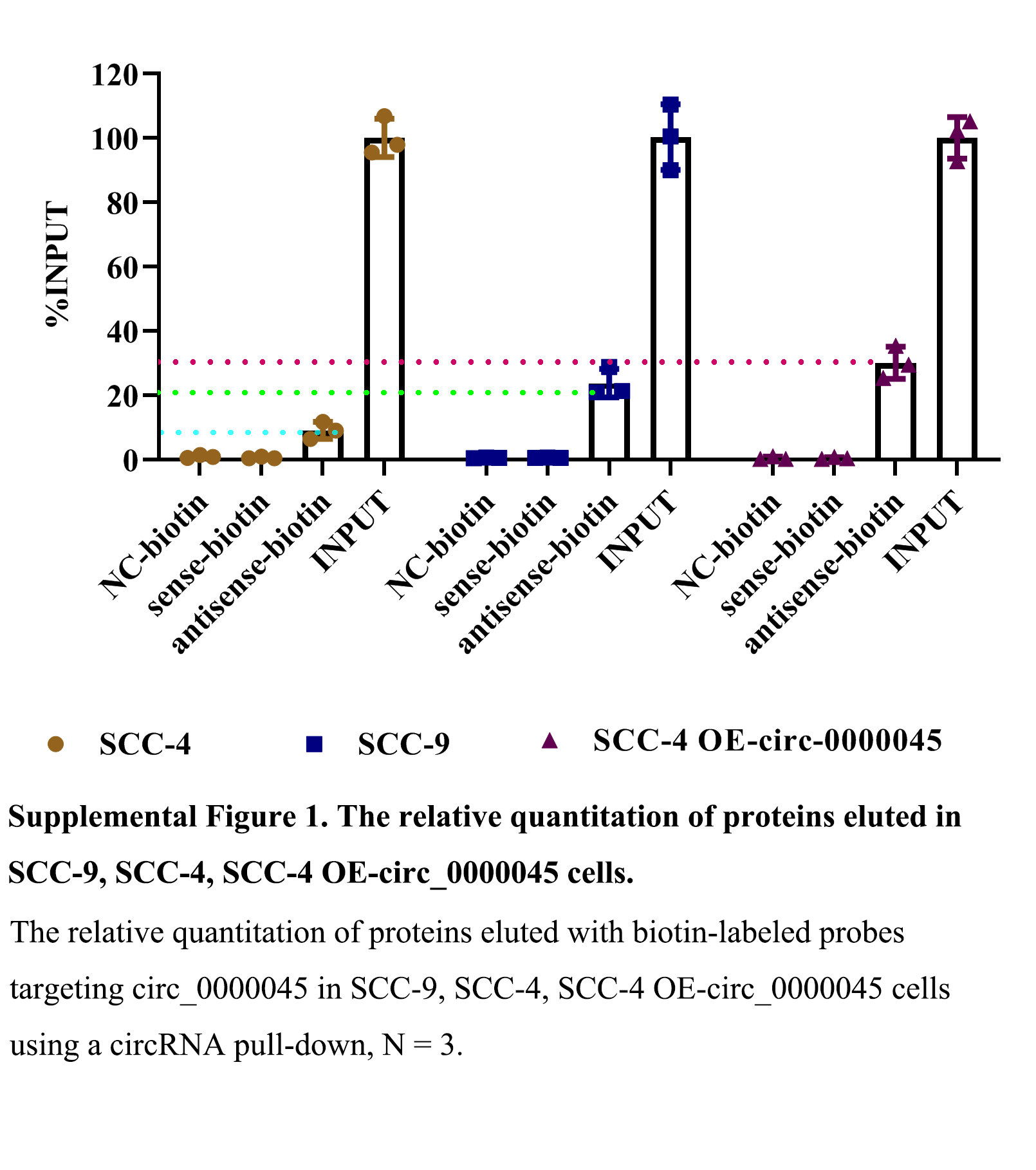

Supplement: Supplementary file 4 — Additional file 4. [file 12885_2022_9880_MOESM4_ESM.tif]

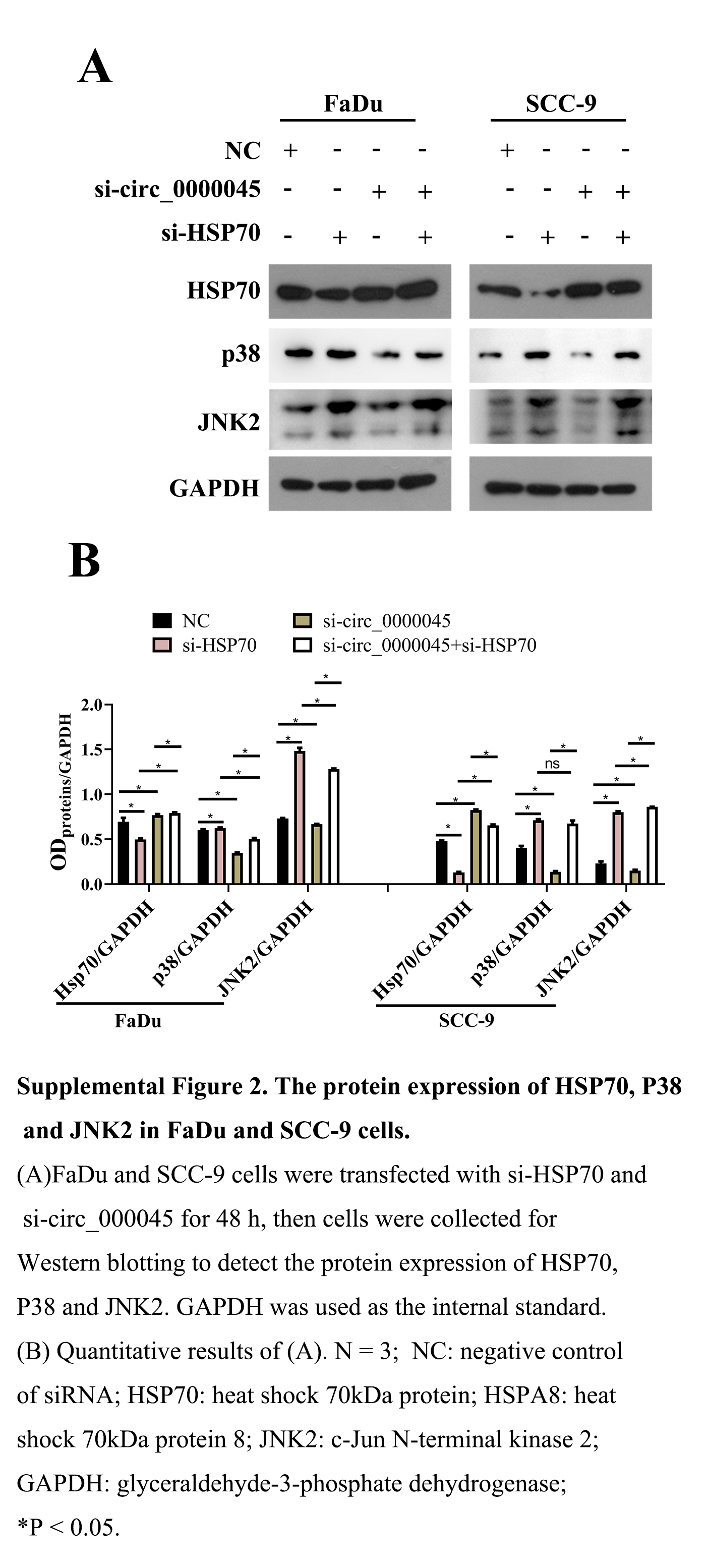

Supplement: Supplementary file 5 — Additional file 5. [file 12885_2022_9880_MOESM5_ESM.tif]
