# Supplementary material for: circ_0000045 promotes proliferation, migration, and invasion of head and neck squamous cell carcinomas via regulating HSP70 and MAPK pathway
Source: BMC Cancer. 2022 Jul 20;22:799. doi: 10.1186/s12885-022-09880-y (PMC9297571; doi:10.1186/s12885-022-09880-y)
Supplement: Supplementary file 6 — Additional file 6. [file 12885_2022_9880_MOESM6_ESM.pptx]

## Slide 1
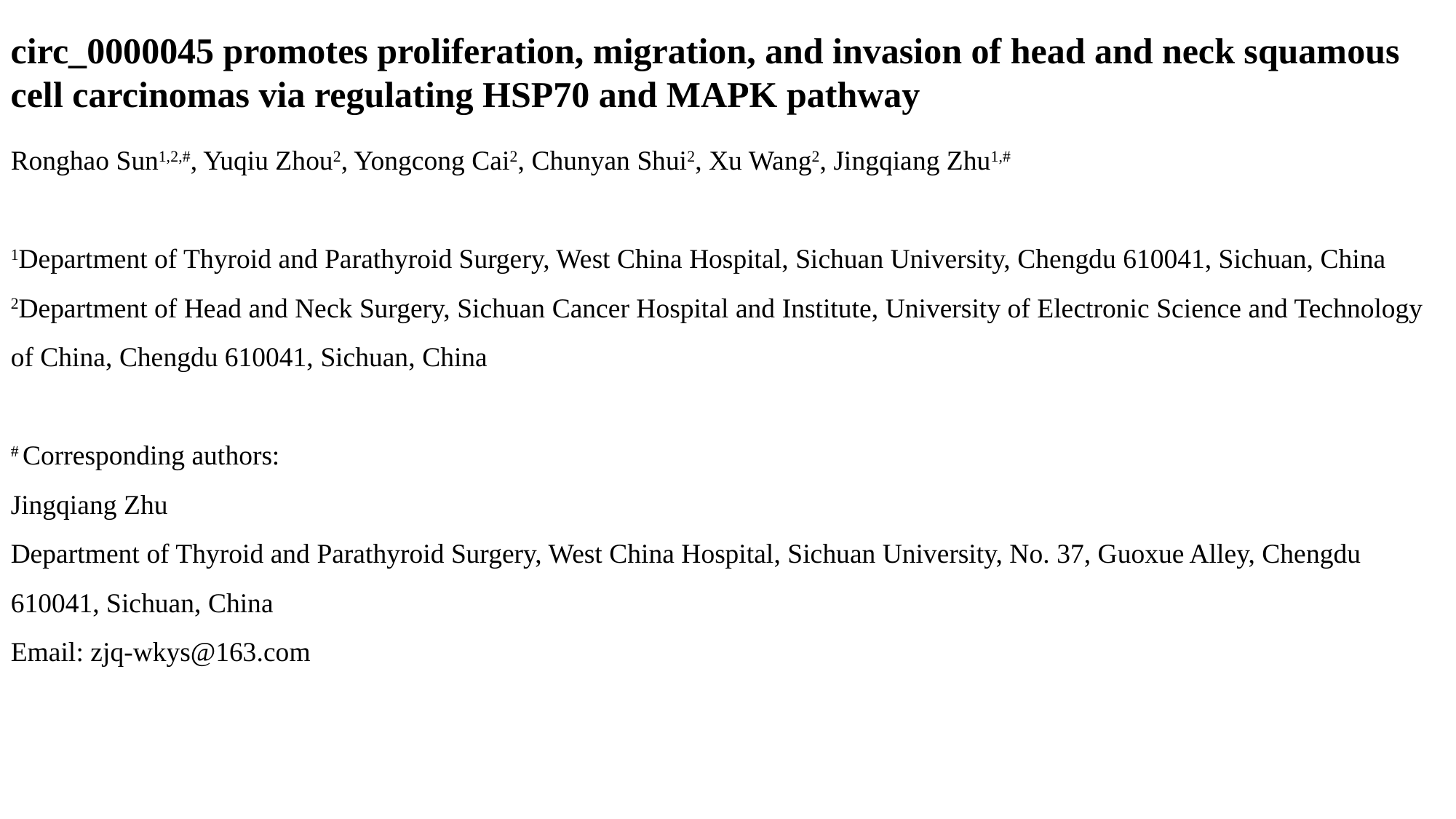

circ_0000045 promotes proliferation, migration, and invasion of head and neck squamous cell carcinomas via regulating HSP70 and MAPK pathway
Ronghao Sun1,2,#, Yuqiu Zhou2, Yongcong Cai2, Chunyan Shui2, Xu Wang2, Jingqiang Zhu1,#
1Department of Thyroid and Parathyroid Surgery, West China Hospital, Sichuan University, Chengdu 610041, Sichuan, China
2Department of Head and Neck Surgery, Sichuan Cancer Hospital and Institute, University of Electronic Science and Technology of China, Chengdu 610041, Sichuan, China
# Corresponding authors:
Jingqiang Zhu
Department of Thyroid and Parathyroid Surgery, West China Hospital, Sichuan University, No. 37, Guoxue Alley, Chengdu 610041, Sichuan, China
Email: zjq-wkys@163.com

## Slide 2
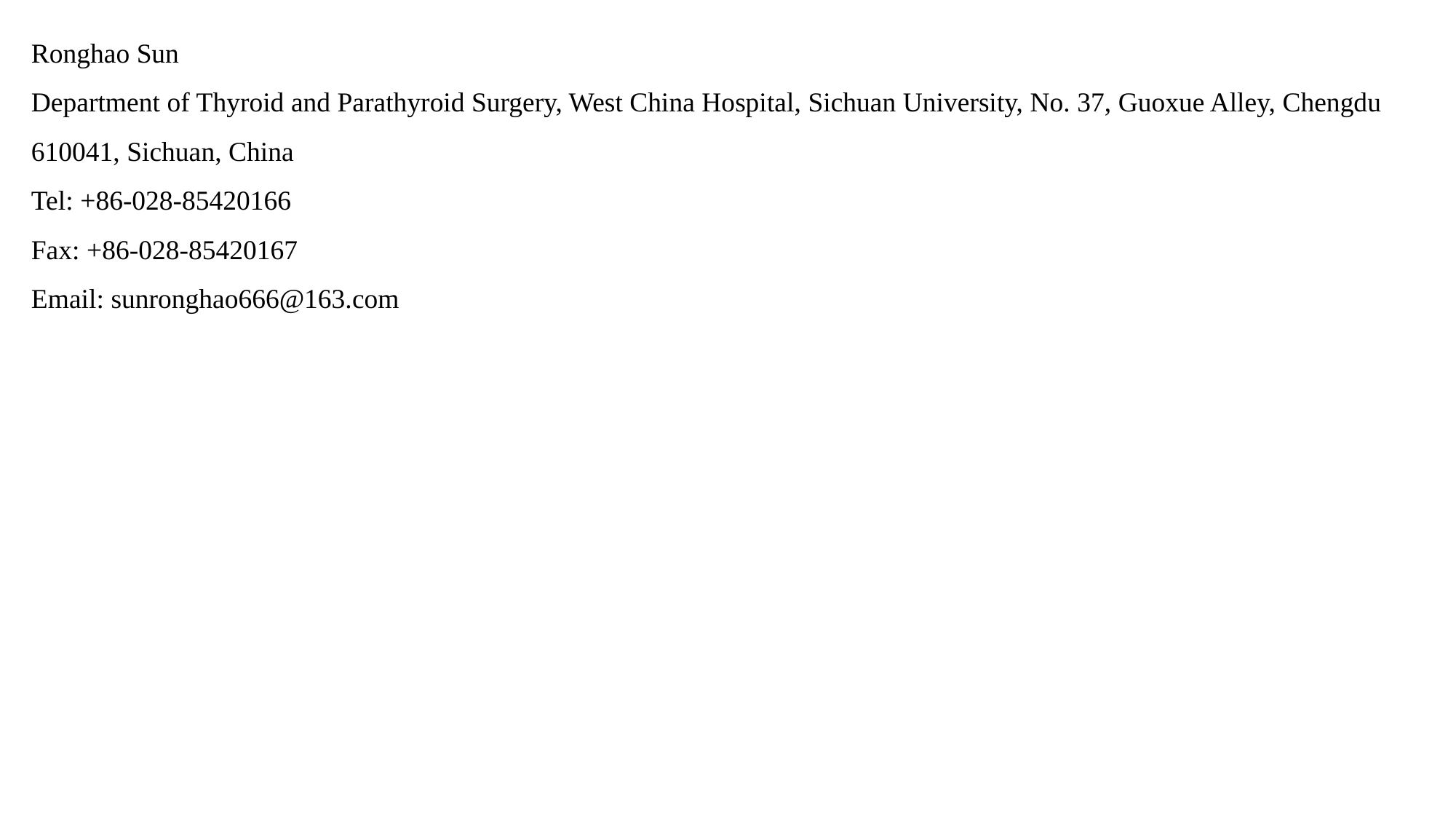

Ronghao Sun
Department of Thyroid and Parathyroid Surgery, West China Hospital, Sichuan University, No. 37, Guoxue Alley, Chengdu 610041, Sichuan, China
Tel: +86-028-85420166
Fax: +86-028-85420167
Email: sunronghao666@163.com

## Slide 3
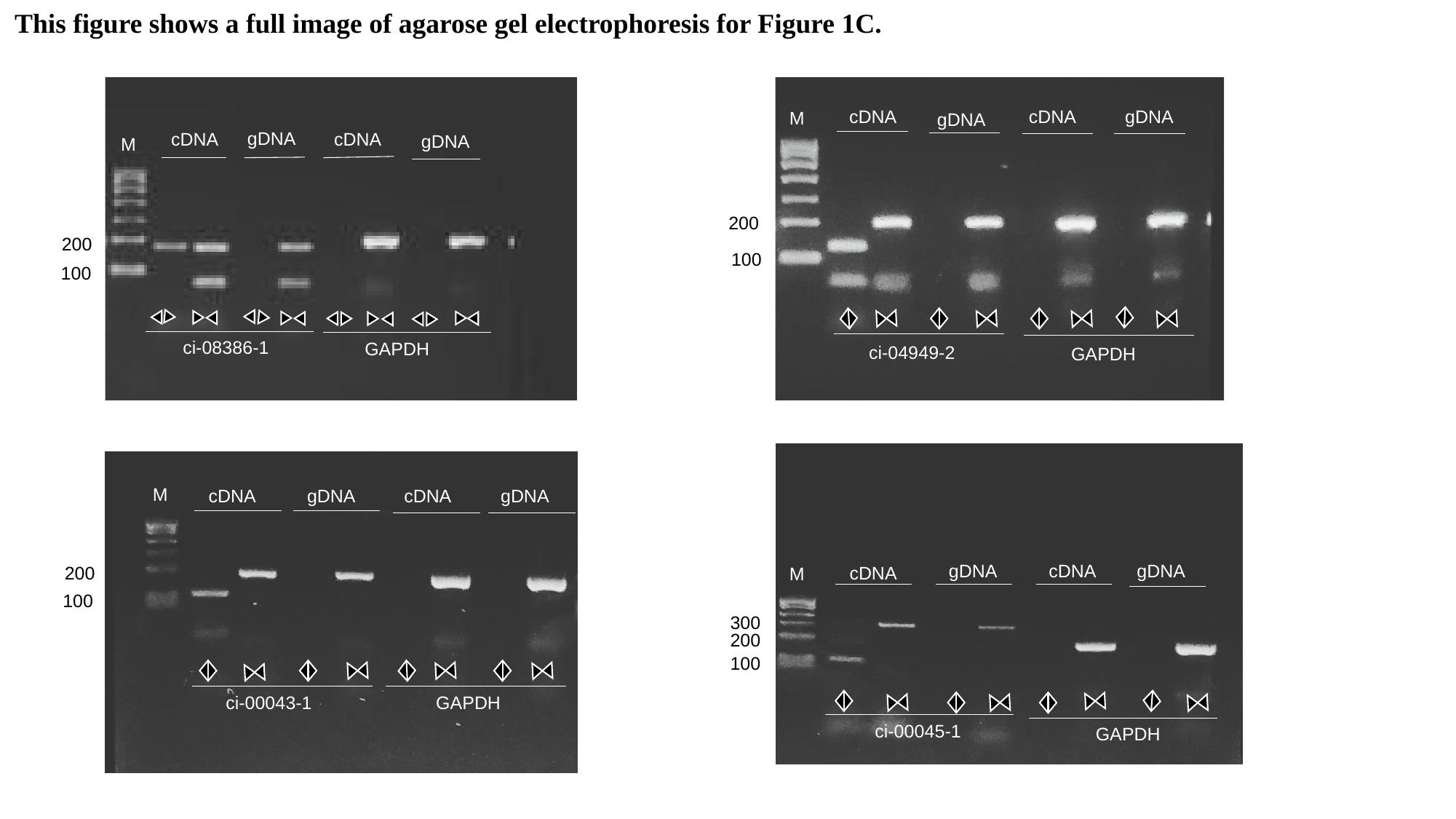

This figure shows a full image of agarose gel electrophoresis for Figure 1C.
cDNA
cDNA
gDNA
M
gDNA
gDNA
cDNA
cDNA
gDNA
M
200
200
100
100
ci-08386-1
GAPDH
ci-04949-2
GAPDH
M
cDNA
gDNA
cDNA
gDNA
gDNA
cDNA
gDNA
200
cDNA
M
100
300
200
100
ci-00043-1
GAPDH
ci-00045-1
GAPDH

## Slide 4
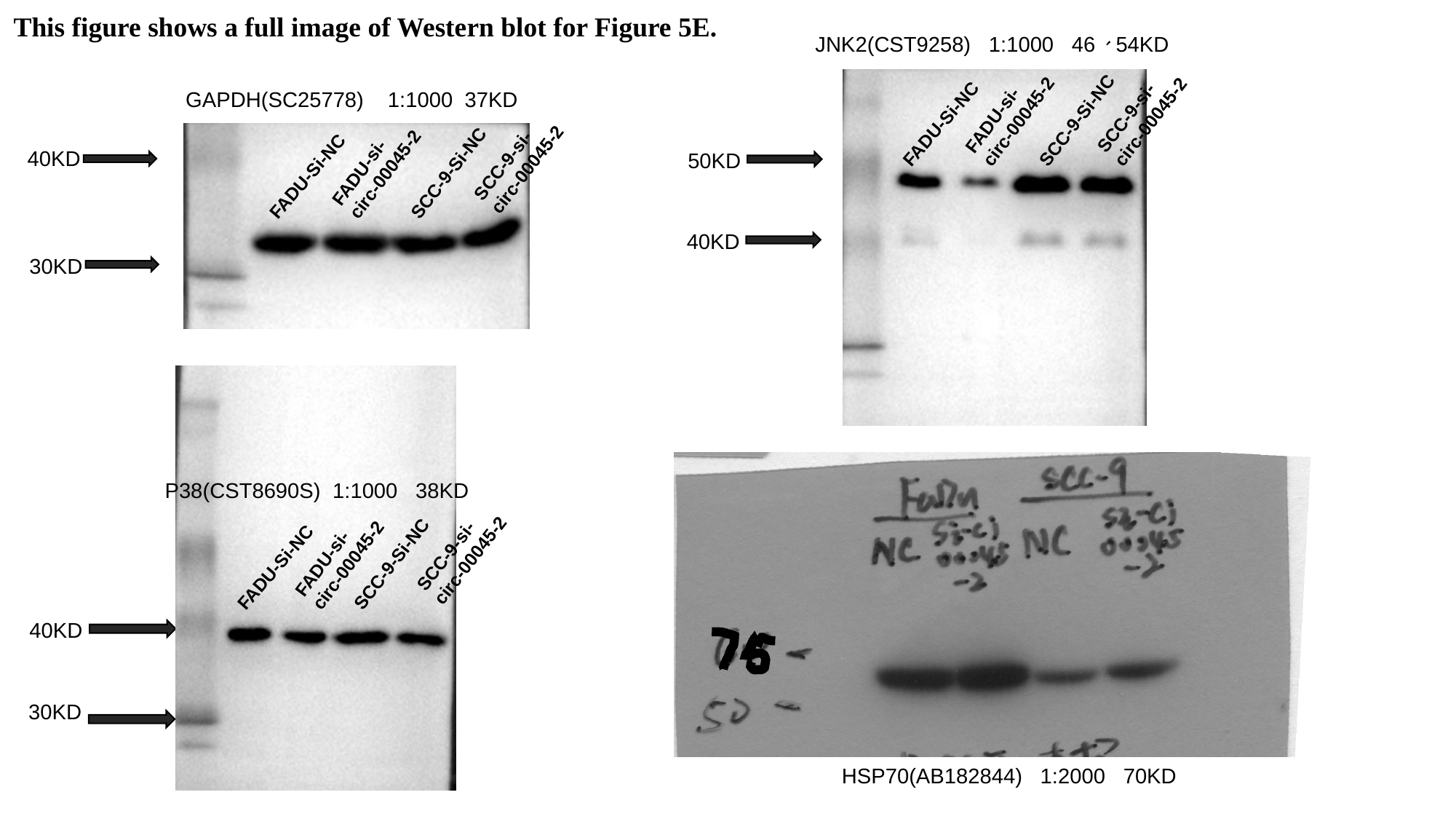

This figure shows a full image of Western blot for Figure 5E.
JNK2(CST9258) 1:1000 46、54KD
GAPDH(SC25778) 1:1000 37KD
FADU-si-
circ-00045-2
SCC-9-si-
circ-00045-2
SCC-9-Si-NC
FADU-Si-NC
SCC-9-si-
circ-00045-2
FADU-si-
circ-00045-2
40KD
50KD
SCC-9-Si-NC
FADU-Si-NC
40KD
30KD
P38(CST8690S) 1:1000 38KD
SCC-9-si-
circ-00045-2
FADU-si-
circ-00045-2
SCC-9-Si-NC
FADU-Si-NC
40KD
30KD
HSP70(AB182844) 1:2000 70KD

## Slide 5
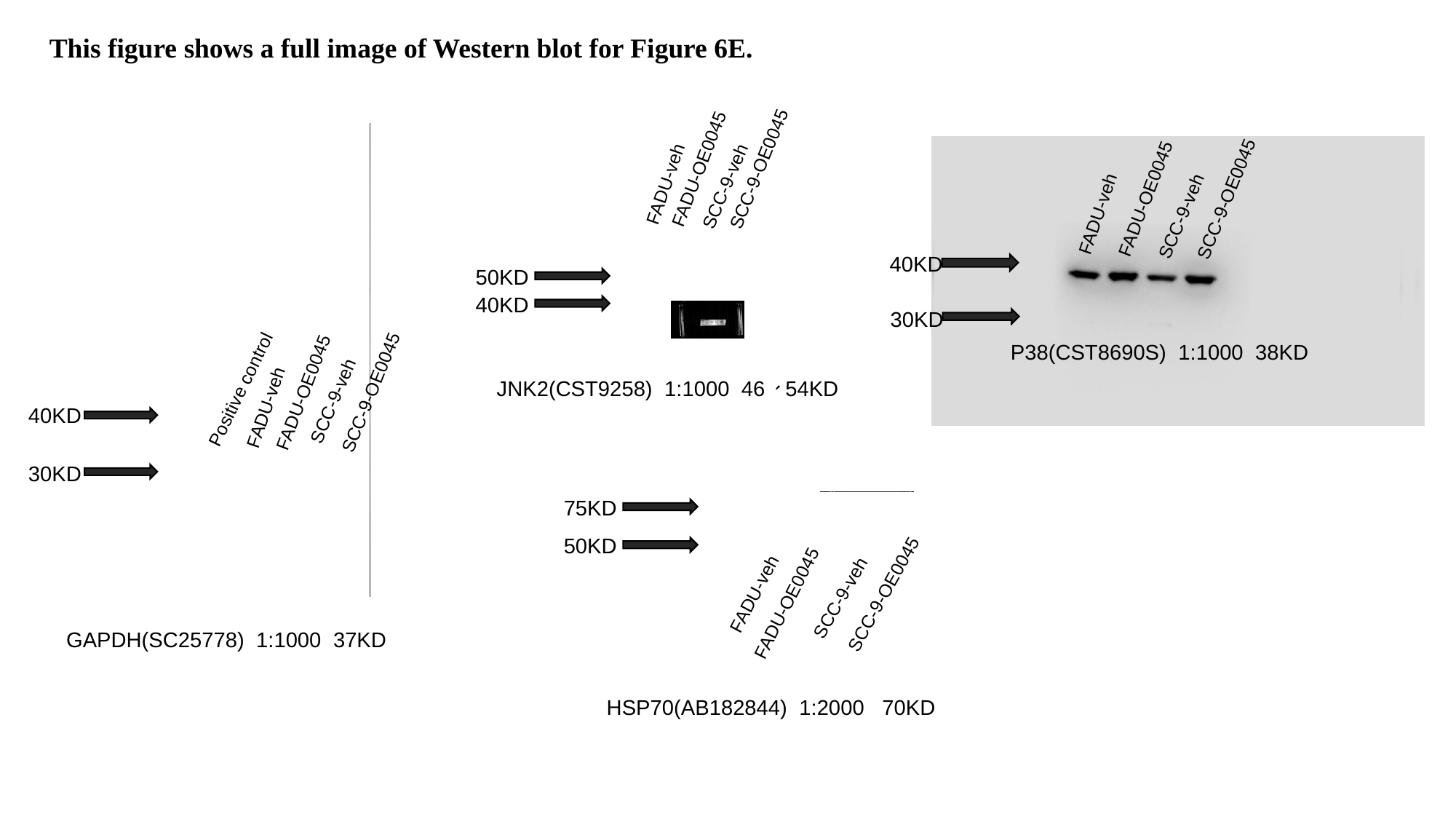

This figure shows a full image of Western blot for Figure 6E.
FADU-OE0045
SCC-9-OE0045
FADU-veh
SCC-9-veh
FADU-OE0045
SCC-9-OE0045
FADU-veh
SCC-9-veh
40KD
50KD
40KD
30KD
P38(CST8690S) 1:1000 38KD
Positive control
JNK2(CST9258) 1:1000 46、54KD
FADU-OE0045
SCC-9-OE0045
SCC-9-veh
FADU-veh
40KD
30KD
75KD
50KD
FADU-veh
SCC-9-OE0045
SCC-9-veh
FADU-OE0045
GAPDH(SC25778) 1:1000 37KD
HSP70(AB182844) 1:2000 70KD

## Slide 6
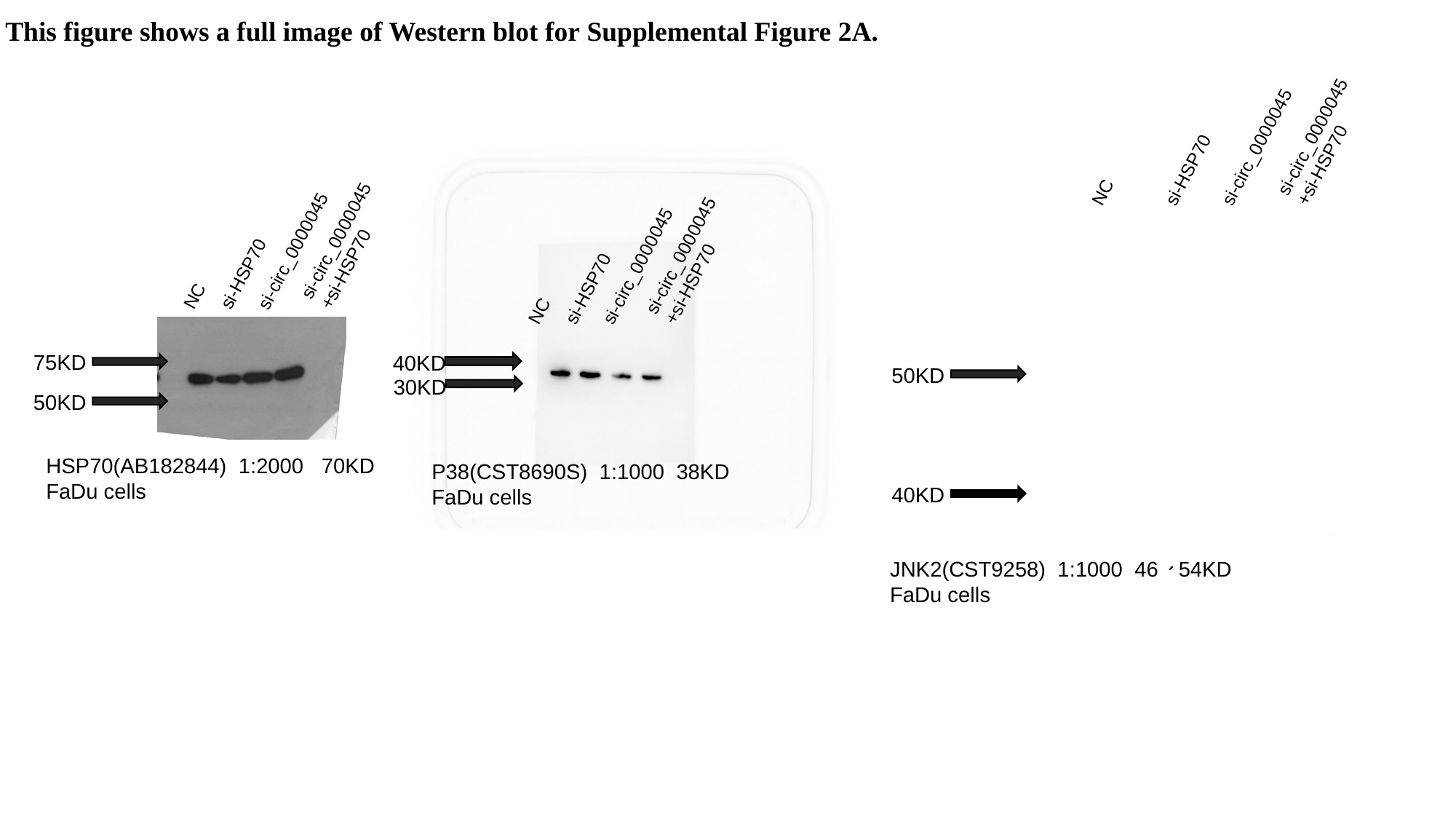

This figure shows a full image of Western blot for Supplemental Figure 2A.
si-circ_0000045
+si-HSP70
si-circ_0000045
si-HSP70
NC
si-circ_0000045
+si-HSP70
si-circ_0000045
+si-HSP70
si-circ_0000045
si-circ_0000045
si-HSP70
si-HSP70
NC
NC
75KD
40KD
50KD
30KD
50KD
HSP70(AB182844) 1:2000 70KD
FaDu cells
P38(CST8690S) 1:1000 38KD
FaDu cells
40KD
JNK2(CST9258) 1:1000 46、54KD
FaDu cells

## Slide 7
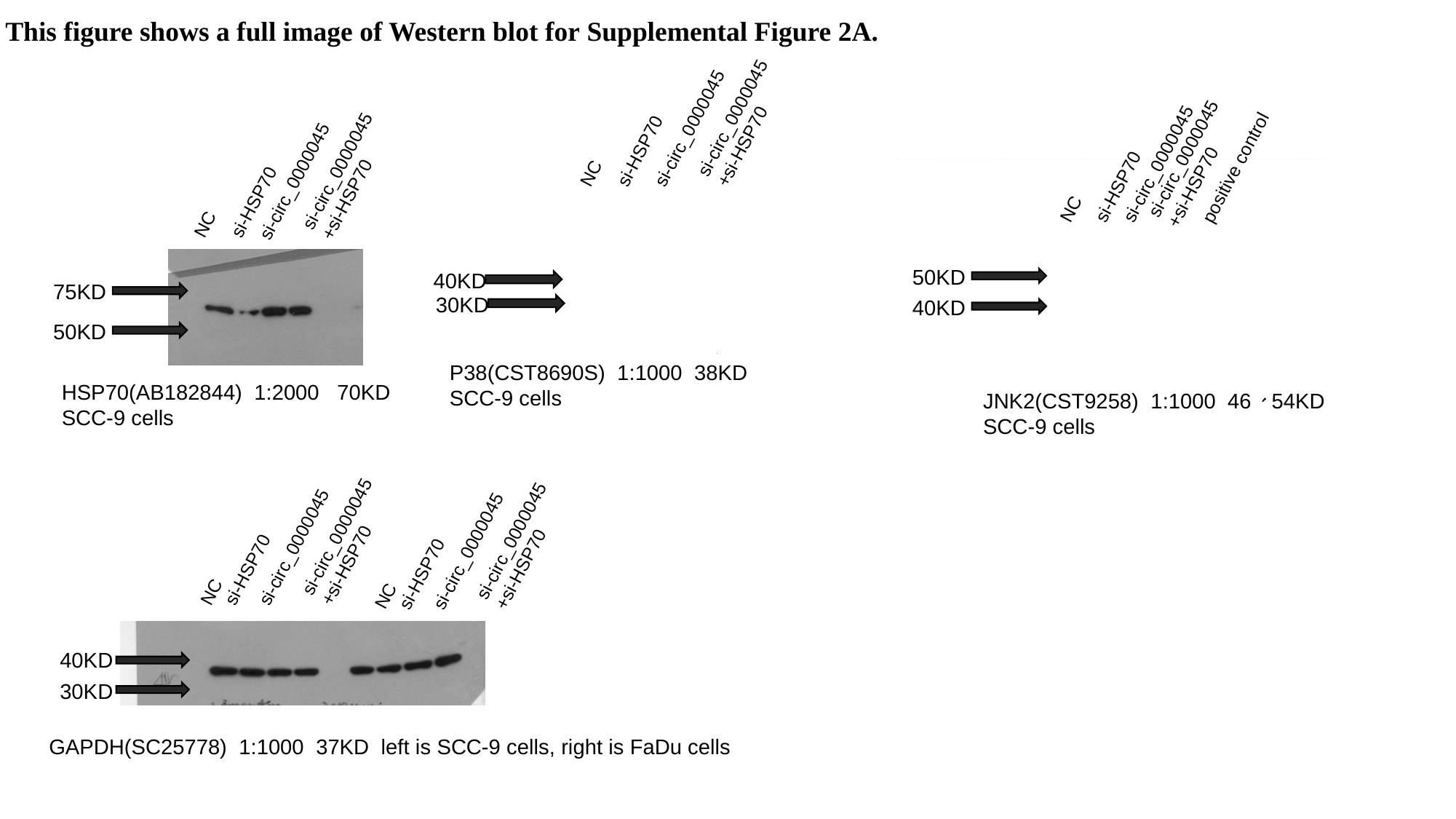

This figure shows a full image of Western blot for Supplemental Figure 2A.
si-circ_0000045
+si-HSP70
si-circ_0000045
si-HSP70
si-circ_0000045
+si-HSP70
si-circ_0000045
si-circ_0000045
+si-HSP70
positive control
NC
si-circ_0000045
si-HSP70
si-HSP70
NC
NC
50KD
40KD
75KD
30KD
40KD
50KD
P38(CST8690S) 1:1000 38KD
SCC-9 cells
HSP70(AB182844) 1:2000 70KD
SCC-9 cells
JNK2(CST9258) 1:1000 46、54KD
SCC-9 cells
si-circ_0000045
+si-HSP70
si-circ_0000045
+si-HSP70
si-circ_0000045
si-circ_0000045
si-HSP70
si-HSP70
NC
NC
40KD
30KD
GAPDH(SC25778) 1:1000 37KD left is SCC-9 cells, right is FaDu cells
